# App-based skills training to reduce problem drinking among adult internet help-seekers: a double-blinded randomized controlled trial

**DOI:** 10.1186/s13722-026-00663-5

**Published:** 2026-03-31

**Authors:** Yaoyu Chen, Anne H. Berman, Claes Andersson, Matthijs Blankers, Olof Molander

**Affiliations:** 1 Department of Psychology, Uppsala University, Uppsala, Sweden; 2 Department of Criminology, Malmö University, Malmö, Sweden; 3 Trimbos Institute, Amsterdam, The Netherlands; 4 Department of Clinical Neuroscience, Karolinska Institutet, Stockholm, Sweden

**Keywords:** Alcohol, Internet help-seekers, Digital interventions, Apps, Randomized controlled trial

## Abstract

**Introduction:**

Problem drinking (PD), defined as hazardous or harmful drinking, constitutes a major health and economic burden worldwide. However, most individuals with PD typically remain untreated for many years as part of the “treatment gap” between prevalence and treatment access. The main objective of this study was to evaluate an unguided, app-based intervention for reducing alcohol consumption, compared to an information control app. Secondary analyses targeted motivation effects, subgroup differences in heavy drinking days, and the proportion of participants with excessive drinking.

**Methods:**

In this two-armed randomized controlled trial (RCT), Swedish-speaking adults seeking help for PD on the Internet were recruited via online ads (*n* = 576). Eligible participants, defined as those who reported PD without significant comorbid conditions, were randomly assigned to one of two unguided interventions, either the “TeleCoach” app (*n* = 293) or the information control app (*n* = 283), and followed up via online questionnaires at six-, 12- and 26-weeks post-recruitment. Participants and researchers were blinded to group assignment at recruitment but not for outcome analyses. The primary outcome was the number of standard drinks consumed per week.

**Results:**

Participants reported drinking a mean of 28 (SD = 18) standard drinks per week at the baseline recruitment measure, and 65% of the participants reported a history of PD for over three years; only 7% of the participants reported less than a year of PD history. Only 26% of the participants had sought help before. A significant within-group reduction in alcohol consumption occurred in both conditions at the six-week follow-up (B = −10.57, SE = 0.83, 95% CI [−12.20, −8.94], *p* < 0.01; Cohen’s d = 0.69), sustained until the 26-week follow-up (B = −11.44, SE = 0.91, 95% CI [−13.23, −9.65], *p* < 0.01, Cohen’s d = 0.67), with no significant differences between the two groups. No adverse events were reported.

**Conclusions:**

The findings, although null, nonetheless supported app-based interventions’ feasibility and potential as a scalable digital approach for reaching individuals who have not previously sought help for PD.

**Trial registration:**

ClinicalTrials.gov as NCT03696888, Oct 5, 2018; analysis plan at https://osf.io/wxfqg, on Dec. 18, 2023 prior to analysis.

**Supplementary Information:**

The online version contains supplementary material available at 10.1186/s13722-026-00663-5.

Problem drinking poses a heavy health and economic burden worldwide. For individuals, problematic consumption of alcohol is a significant risk factor for injury, various cancers, and chronic diseases [1]. According to meta-analysis, the average cost attributable to alcohol use amounts to 1306 USD per adult, or 2.6% of the gross domestic product among most high-income and some middle-income countries included in the analysis. In addition to direct costs (e.g. crime, health care, traffic accidents), indirect costs (e.g. productivity losses) are estimated to be even higher, up to two-thirds of the total costs [2]. In addition, individuals with lower income and greater societal marginalization are especially vulnerable to the disease burden for every unit of alcohol consumed [3]. Reducing problematic drinking through effective and scalable alcohol interventions could have considerable economic and social justice impacts.

Problem drinking (PD) refers to a behavioral pattern of continued hazardous alcohol use or directly harmful alcohol use that leads to adverse consequences, such as physical harm or impaired daily functioning. PD is a pivotal aspect of Alcohol Use Disorders (AUD) [4, 5]; however, individuals with PD often do not seek healthcare providers due to a lack of treatment accessibility and/or affordability, lack of problem awareness, stigmatization, and the desire to cope alone [6–9]. A recent study estimated that, globally, only one in six people with AUD received any treatment for alcohol use, and the treatment rate is even lower, at 9.3%, in low and lower-middle-income countries [10]. For individuals diagnosed with AUD, reduced alcohol consumption is associated with a substantially lower mortality risk, and achieving abstention can lead to a further reduction in mortality [11].

Digital interventions are a promising approach to deliver cost-effective, accessible, and highly scalable interventions for mental health and health behavior change [12]. Once developed and deployed, unguided digital interventions can scale up to any number of users with minimal extra cost per person [13]. A recent review found a moderate aggregate effect size for mental health digital interventions, both with and without human guidance, in low- and middle-income countries [14], supporting the scalability of using digital interventions to meet global health challenges. In 2023, over half of the global population owned a smartphone [15], and smartphone ownership has reached at least 40% of the population in low-income countries such as Kenya, Nigeria, and Indonesia [16]. This means digital interventions optimized for mobile devices can potentially reach individuals in places with limited healthcare options. Interventions delivered by text messages can reach all types of mobile phones. However, a text-message approach can become unfeasible when the intended users have limited network access. Mobile apps, in contrast, can offer treatment content that is immediately available in one’s pocket [17].

Many digital interventions for PD are unguided brief alcohol interventions, which lead to a small but significant reduction in alcohol consumption; human-guided interventions produce larger effect sizes but reduce potential scalability [18]. A recent meta-analysis of a wide range of digital interventions for individuals with hazardous and harmful alcohol use showed that digital interventions were to some extent effective in reducing alcohol consumption by small quantities, as measured in grams per day, drinking days per week, heavy episodic drinking and scores on the Alcohol Use Disorders Identification Test (AUDIT), in comparison to no or minimal treatment, as well as in-person treatment [19].

Recent research on apps to reduce PD includes Oldham et al. [20], who evaluated the Drink Less app against usual digital care (NHS alcohol advice webpage, and Cunningham et al. [21], who compared a full intervention app against an education-only control. The pattern of findings in these studies, among help-seekers in the general population, suggests the limited effectiveness of apps with enhanced content compared to educational content alone. Two apps targeting patients during and following residential or rehabilitative treatment that have shown positive results include the A-CHESS and UControlDrink apps. Both A-CHESS and UControlDrink app users demonstrated fewer problem drinking days per week than participants with only treatment as usual [22, 23].

One app-based intervention that has shown promising effects among non-treatment-seeking university students is TeleCoach, a web app-based, self-guided alcohol intervention integrating personalized feedback, relapse prevention, and strategies for emotion regulation [24, 25]. Developed on the basis of an Interactive Voice Response (IVR) intervention [26], the TeleCoach app content was originally intended for a range of adult populations, but was first evaluated among Swedish university students who reported excessive drinking, defined as > 9 drinks/week for women and >14 for men by the Swedish National Board of Health and Welfare [27]. In this group, the TeleCoach app was associated with reduced drinking quantity at 7-week follow-up and reduced frequency of drinking at 7- and 14-week follow-ups compared to assessment-only controls, with the odds of eliminating excessive consumption nearly twice as high in the TeleCoach group [25]. Indeed, TeleCoach was particularly effective for a subgroup of individuals with a frequent-heavy drinking pattern, who reported averaging 4.6 drinking days per week and consumed 16.4 drinks weekly – significantly higher than the study average of 8.9 drinks [24]. Compared to assessment-only controls, this group showed robust reductions in drinking days with TeleCoach access. Higher baseline motivation to reduce drinking was furthermore associated with greater reductions in consumption over time among app users compared to assessment-only controls, suggesting that more motivated users might benefit more from apps that provide extensive content and skills training.

The current trial built on previous research by extending TeleCoach evaluation from the population of university students to adults from the general population seeking help on the internet for problem drinking, termed “internet help-seekers” in this article. The main objective of this trial was to compare the intervention app to an information-only control app, in a two-armed, double-blinded randomized controlled trial with the number of drinks consumed in the past week defined as the primary outcome. Secondary analyses explored the effects of motivation to change alcohol consumption, as well as identifying a frequent-heavy sub-group, who reported drinking large quantities on several days a week [24], and analyzing related app effects on their drinking pattern.

As our primary hypothesis (PH), we postulated that the mean number of standard drinks in the past week would be lower in the intervention group than in the control group at all follow-ups. Four secondary hypotheses (SH1-4) were formulated for each of the follow-ups, as follows: Higher individual motivation to change in both the intervention and control groups would be associated with a lower past-week quantity of standard drinks at all follow-ups (SH1); the association between higher readiness to change and lower past-week drinking quantity would be greater in the intervention group than in the control group (SH2); a group of frequent-heavy drinkers would be identified, where the intervention group would show a pattern of fewer heavy drinking days compared to those in the control group (SH3); and the proportion of participants with excessive drinking, i.e., drinking more drinks per week than recommended by national guidelines in Sweden would be lower in the intervention group than in the control group following intervention (SH4),

## Methods

This study was a two-armed, double-blind, placebo-controlled, parallel-group trial with a 1:1 allocation ratio between the intervention and control groups. Participants were informed that they would be assigned to one of two conditions, both aiming to support their efforts to decrease hazardous or harmful alcohol consumption. Participants were randomized to either the intervention or the control version of the app using a 20 × 50 block randomization and blinded to their allocation status. Based on the randomization outcome, the participant automatically landed in either the intervention or the control version of the app. Researchers were blinded to randomization results until after study completion.

## Sample size and recruitment rationale

A pre-trial power analysis indicated that a total of at least 100 participants per group at 26 week follow-up would be required to detect a between-group effect of *d* = 0.24 at 80% power with an α = 0.05 [18, 28]. Based on the standard deviation of the primary outcome observed at follow-up in the pilot study (SD = 10.8 standard drinks/week), this effect size translates to a difference of approximately 2.6 standard drinks per week between groups. Given high attrition in trials targeting internet help-seekers [18, 29], as well as uncertainty regarding TeleCoach app effects in the adult population, as opposed to the younger student population, the target for this trial was to recruit at least 1000 participants at the screening stage, with the expectation that high attrition would occur at follow-up measures; recruitment was thus stopped after reaching 1000 participants at the screening stage. All participants were recruited through Swedish-language advertisement on Google AdWords, where searches for relevant keywords (e.g., “alcohol problems”, “alcohol help”, or “alcoholism” in Swedish) triggered the appearance of a study recruitment advertisement on the search results page. Clicking on the recruitment ad led the user to an online screening survey page which starts with study information and consent.

## Inclusion and exclusion criteria

The study was designed to select adults with PD without significant comorbid conditions. The initial study protocol stipulated two inclusion criteria [1]: excessive weekly alcohol consumption according to then-applicable public health guidelines in Sweden (>9 drinks per week for women and >14 drinks per week for men) [27]; and [2] fulfilling DSM-5 (APA, 2022) criteria for mild to moderate alcohol use disorder (AUD) but not severe AUD (i.e., ≤6 AUD criteria) [4]. In effect, the initial inclusion criteria required potential participants to engage in harmful alcohol consumption without meeting the DSM-5 criteria for severe AUD, and this led to the exclusion of most potential participants with excessive drinking.

After 2 weeks of recruitment, the inclusion and exclusion criteria were therefore revised as follows: (a) being at least 18 years of age with (b) signs of at least hazardous alcohol use, defined as scoring ≥ 6 (women) or ≥ 8 (men) on the Alcohol Use Disorders Identification Test (AUDIT) [30]. The gender-specific cut-offs were based on a psychometric study of AUDIT in Sweden, demonstrating its sensitivity to gender differences [31]. Individuals satisfying any of the following conditions were excluded: (a) severe depression, as indicated by > 30 on the Montgomery Åsberg Depression Rating Scale (MADRS-S) [32]; or (b) suicidal ideation, as indicated by scoring > 4 on question 9 of the MADRS-S; or (c) comorbid drug use disorder, as indicated by scoring ≥ 8 on the Drug Use Disorders Identification Test (DUDIT) [33, 34]. To incentivize participants, they were informed that those who completed all follow-ups at 6, 12, and 26 weeks would be eligible for an iPad lottery.

## Procedure

Included participants were thus those who consented to the trial, completed the screening survey, and fulfilled all inclusion and exclusion criteria following completion of the baseline survey that followed screening. Once included, participants were automatically randomized to access either the intervention or the control app with a unique link. Participants were informed that they could access the app via a mobile phone or desktop browser; the app was thus programmed as web-based rather than being a native app. No further instructions were provided regarding how to use the app, when, or how long to use it; participants were free to engage with the app as they wished. Then, the survey email system scheduled follow-up surveys at 6-, 12-, and 26-weeks following inclusion. Participants who did not respond were sent reminder emails every week until participants responded, chose to opt out or until the study concluded. Some participants thus received multiple reminders, due to a limitation in the digital survey used. Participants who wanted to be removed from continued reminders could do so by contacting the principal investigator. Participants had access to the app throughout the entire follow-up period, regardless of whether they completed follow-up measures.

## Ethics

This study involved human participants who provided sensitive personal health-related data. The Swedish Ethical Review Authority reviewed and approved this study (approval number 2016/1088–31, approved June 28, 2016; amendment number 2018/2569–32, approved December 28, 2018) prior to recruitment. All participants provided their informed consent via the online survey.

## Measures

The primary outcome was the change in total number of standard drinks consumed in the past week reported by participants with the Timeline Follow-back (TLFB) [35]. The secondary outcomes, e.g., estimated blood alcohol concentration (eBAC), required data from the Daily Drinking Questionnaire (DDQ) [36, 37]. Therefore, the TLFB and DDQ were included in all follow-ups after screening and baseline measures. The 26-week follow-up included all screening and baseline measures. Self-reported use of other treatments during the study, along with user satisfaction, was assessed at the 26-week follow-up. While all baseline measures were repeated at the 26-week follow-up, only those relevant to this study’s hypotheses were included in the analysis. All measurement instruments are described below.

### Primary outcome measure

**Timeline Follow-back (TLFB).** The TLFB [35] asks participants to retrospectively report their substance use quantity for each day in a specific time window. The TLFB has shown good psychometric properties [38–40], including when used for self-administration online [41, 42], making it a commonly used quantitative instrument for substance consumption [43]. This study used the TLFB to quantify total alcohol consumption in the past seven days at baseline and at each follow-up. The questionnaire included the standard-drink equivalent of common alcoholic beverages (e.g., beer, spirits, wine), which in Sweden is equivalent to 12 grams of alcohol. Using these examples, participants were asked to report their drinking quantity on the previous day, then going back one day at a time until reaching the seventh day of the past week. The 7-day TLFB has shown indications of being more accurate than TLFB versions that cover longer intervals [44].

### Secondary outcome measures

**The Daily Drinking Questionnaire.** The DDQ asks participants to report their drinking pattern for a typical week in the past month [36, 37] as well as their gender and weight to allow for the calculation of their estimated blood alcohol concentration (eBAC). The DDQ thus provides detailed information about each participant’s drinking quantity, frequency, binge occasions, and mean and peak eBAC. Quantity is a count of the total number of standard drinks consumed in a typical week of the past month. Frequency is a count of days with at least 1 standard drink consumed. A binge occasion is a day when a participant drank 4 or more standard drinks for women or 5 or more standard drinks for men [45]. The eBAC is calculated based on the Widmark formula [46]. The average eBAC is the mean of daily eBAC in the typical 7-day period reported. The peak eBAC is calculated from the day the respondent reported drinking the most in the past month.

**Readiness Ruler (RR).** The RR is a visual analogue scale that measures motivation by asking participants to rate their readiness to change their drinking habits on a scale of 0 (i.e., “I am not ready to change my drinking habits) to 10 (i.e., “I am extremely ready to change …”). The scale is often used as a clinical tool in Motivational Interviewing [47], as well as in research to assess readiness/motivation for change [48–51].

### Screening and enrollment measures

**Alcohol Use Disorders Identification Test (AUDIT).** The 10-item AUDIT is a self-report screener for problem drinking (hazardous or harmful) developed by the WHO [30]. The screener asks about drinking frequency, quantity, and alcohol-related problems. The first 8 items are scored from 0 to 4. The last 2 items, which ask about harm to self or others and concerns expressed by others, are scored 0 (“No”), 2 (“Yes, but not in the last year”), or 4 (“Yes, during the last year”). Total scores range from 0 to 40, with higher scores indicating greater severity. The psychometric properties have been validated for primary care use in Sweden [31]. Meta-analytic evidence across 342 studies indicates good internal consistency with a Cronbach’s α of 0.82 [52].

**Montgomery-Åsberg Depression Rating Scale (MADRS-S).** Depression and suicidal ideation were measured with the self-report version of the MADRS [32, 53]. Participants were asked to rate their state of mind on a Likert scale ranging from 0 to 6. Total scores range from 0 to 54, with higher scores indicating greater depression severity. A total score of 30 was used as the threshold for severe depression [54]. Item 9 of the scale measures suicidal ideation; scoring more than 4 points on this item was used as the indicator for suicide risk. The MADRS-S has demonstrated satisfactory internal consistency with a Cronbach’s α of 0.84 [55] and good psychometric properties when administered online [56].

**Drug Use Disorders Identification Test (DUDIT).** The 11-item DUDIT measured participants’ problematic drug use disorder [33, 57]. The questionnaire specifically focuses on “drugs other than alcohol” with a list of common illicit drugs provided for reference. Item 1–9, which are scored 0–4, measure the frequency and functional impairments of drug use. Items 10–11, which are scored 0, 2, or 4, measure past harm to self or others and concerns expressed by others. Multiple psychometric studies have demonstrated that the DUDIT is robust regarding reliability and validity [58], with internal consistency ranging from Cronbach’s α of 0.80 to 0.93 [33].

### Baseline descriptive measures

**Screening for Alcohol Use Disorders (AUD).** In addition to the AUDIT, participants also responded to a 13-item DSM-5 criteria-based questionnaire for alcohol use disorders. The screener was adapted from a validated Swedish translation with a Cronbach’s α of 0.86 [59] of a DSM-based diagnostic screener for AUD among college students [60]. Out of 13 items, 11 items correspond to DSM-5 criteria. The two other items accounts for potential interpretation differences regarding tolerance and withdrawal. Each item is rated as present or absent; total score ranges from 0 to 12.

**The Penn Alcohol Craving Scale (PACS).** The 5-item PACS measures alcohol craving via self-report of craving frequency, intensity, and duration, the ability to resist craving, and overall craving in the past week. The item responses are scaled from 0 (“Never”) to 6 (“Nearly all of the time”). Total scores range from 0 to 30, with higher scores indicating more intense craving. The PACS is a valid and reliable measure (Cronbach’s α = 0.92) and has been shown to predict the risk of relapse [61].

**Generalized Anxiety Disorder (GAD-7).** The GAD-7 is a 7-item self-report screener for Generalized Anxiety Disorder (GAD) as defined by DSM-IV diagnostic criteria [62]. The screener asks about the frequency of participant experiences of the seven symptoms of GAD in the past two weeks on a scale of 0 (“Not at all”) to 3 (“Nearly every day”). Total scores range from 0 to 21. The GAD-7 is widely used and well-supported for its reliability and validity with a Cronbach’s α of 0.92 [63]. Here, we adopted a cutoff score of 8 for anxiety disorders as recommended by a diagnostic meta-analysis [64].

**The Alcohol Abstinence Self-Efficacy Scale (AASE).** The AASE is a 20-item self-report questionnaire that assesses abstinence efficacy based on Bandura’s conceptualization of self-efficacy [65]. The scale asks participants to rate their confidence in their ability to abstain from alcohol across 20 high-risk situations in four categories/subscales: negative affect, social positive, physical and other concerns, and withdrawal and urges. Each item is rated on a 5-point Likert scale from 1 (not at all) to 5 (extremely), indicating confidence to abstain from alcohol in that situation. Higher scores indicate greater abstinence self-efficacy (Spearman–Brown *r* = 0.95, 65).

## App format and content

The intervention and control apps had the same login procedure, visual style, and interaction logic; the only differentiating factor was the content of the intervention. The intervention app, TeleCoach, focuses on self-monitoring of alcohol consumption and skills training for abstinence. The control app offered a brief assessment and psychoeducational information about excessive alcohol consumption. Usage data was automatically collected for both apps.

**Fig. 1:**
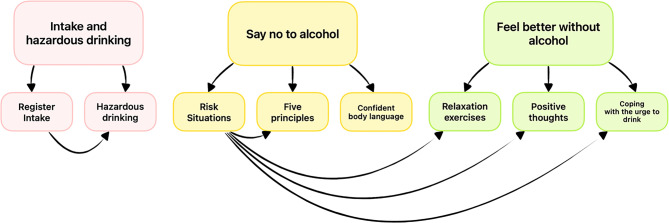
TeleCoach app component and content unit structure

**TeleCoach App.** The intervention app, TeleCoach, is a personalized skills training app for alcohol abstinence. The app is currently available in English, Arabic, Chinese, and Swedish at https://telecoach.vercel.app/. Figure 1 shows a detailed flow chart outlining the app’s navigation logic. The app is described in more detail in prior publications [24, 25, 28] but is described briefly here. It includes three main components: “Intake and hazardous drinking,” “Say no to alcohol,” and “Feel better without alcohol.” Each component comes with two or three content units. Components and units are arranged in the order conjectured to appeal to users, but users are free to start with any of the modules. In the first component, “Intake and hazardous drinking,” participants are asked to assess their drinking quantity in the past 7 days with TLFB so that the app can provide personalized feedback based on their most recent drinking quantity. This feedback, based on automatic summation of participants’ past 7-day drinking, informs participants if they have consumed more than the recommended amount (i.e., more than nine drinks per week for women or 14 for men). The same feedback also suggests clicking to learn more about risks associated with different drinking quantities. The second component, “Say no to alcohol,” incorporates the AASE to identify situations where participants are most and least likely to drink. Based on the results, participants are directed to other units that target those high-risk situations. For instance, if a participant reports that they are most likely to drink when they are happy with others, the results suggest that they visit the “Five Principles” psycho-education unit under the same component. This page offers the user five ways to refuse alcohol offers [66], followed by a questionnaire regarding their self-efficacy after completing the exercise. The “Say no to alcohol” component contains two units that introduce skills for participants to refuse alcoholic beverages more effectively in social settings. The “Feel better without alcohol” component introduces guided relaxation, cognitive modification, and an urge surfing exercise to help participants reduce their alcohol use.

**Control App.** The control app offered risk-related psychoeducation about hazardous drinking, such as identifying early signs of risky drinking, signs of hazardous consumption, and relevant negative consequences. Unlike the intervention app, the control app did not offer any personalized components; only psychoeducation was offered. The information provided in the control app was adapted from a prior study [67] and used with permission. The content in the control app is not publicly available in app form.

## Statistical analysis

All analyses were computed with R statistical language [68]. The primary outcome, the total number of drinks in the past week, showed a positive skew and a long tail across all follow-ups (skewness ≥ 1.8; see Supplemental Fig. 1). This distribution pattern is often expected of behavioral count outcomes [69]. To account for the non-normal distribution, we used three robust linear mixed-effects models with R package *robustlmm* [70] to estimate (a) the effects of treatment condition over time, (b) the effects of readiness to change over time, and (c) the interaction between treatment conditions and readiness to change over time. Age, gender, having sought help before, anxiety, and depression were entered as time-invariant covariates for all three models. Individual participants were modeled as the random effect. The time variable was coded as a categorical variable with four levels: baseline, 6, 12, and 26 weeks. Standardized effect sizes (Cohen’s d) were computed by dividing model-estimated contrasts by the residual standard deviation of the robust mixed-effects model (i.e., model-based Standardized Mean Differences); within-group effects compare each follow-up with baseline. Since a potential zero-inflation was also observed, findings from the robust linear mixed models were further validated against zero-inflated negative binomial mixed models post hoc with the *glmmTMB* package [71].

Analyses for the primary hypothesis and the secondary hypotheses related to motivation effects were conducted following the intention-to-treat principle, with all participants analyzed according to their randomized group assignment. Missing data were handled by robust linear mixed models [70], which estimate parameters via likelihood-based methods using all available observations from each participant without requiring imputation. Like full information maximum likelihood and multiple imputation, mixed-effects repeated measures approaches produce valid inferences under the missing at random assumption and have been shown to yield less biased treatment effect estimates and standard errors compared with last observation carried forward or single-imputation methods [72, 73]. Simulation studies in clinical trials supported that likelihood-based mixed models maintain appropriate Type I error rates in the presence of dropout, whereas methods such as last observation carried forward and worst-case imputation produce substantially inflated error rates [73, 74]. Therefore, no additional imputation was considered necessary for the primary analyses [75, 76].

For analysis of motivational levels, dichotomization of RR outcomes were data-driven and recoded as low (≤8) or high (>8) due to skewness. For the sub-group analysis, we constructed Hidden Markov Models (HMMs) with the *depmixS4* R package [77]. The model was specified to predict three drinking levels for each day: “abstinent” (0 drinks), “moderate” (1–3 drinks for women and 1–4 for men per day), and “heavy” (4 or more drinks for women and 5 or more for men per day). The drinking level variable was recoded from the estimated standard glasses for the seven days in a typical week assessed by the DDQ. Days with missing data were excluded from analysis. Models were iteratively fitted with 8 to 16 states and the model with the lowest Akaike Information Criterion (AIC) value was selected. Another linear mixed model was constructed to check if frequent-heavy drinkers in the treatment condition had fewer drinking days than those in the control condition. This model was specified to predict the total DDQ-estimated drinking quantity with a time-condition interaction term and the random effect modeled at the individual level.

To align with the previous RCT study among university students [25], we used Pearson’s chi-squared test to compare the proportion of participants with excessive drinking across all follow-ups. In addition, we also cross-validated the results with different analytical strategies: we examined DDQ and TLFB results using the old and new Swedish national guidelines for excessive drinking (old: over 9 drinks per week for women or over 14 drinks per week for men; new: over 10 drinks for anyone; The Swedish National Board of Health and Welfare, 2023), both with and without multiple imputation for missing data for the Pearson’s chi-squared test (see Supplemental Table 1).

The study included a nested pilot study, which has been published [28]. The current analysis plan was registered at https://osf.io/wxfqg before conducting the comprehensive data analysis reported here.

## Results

### Sample characteristics

**Fig. 2:**
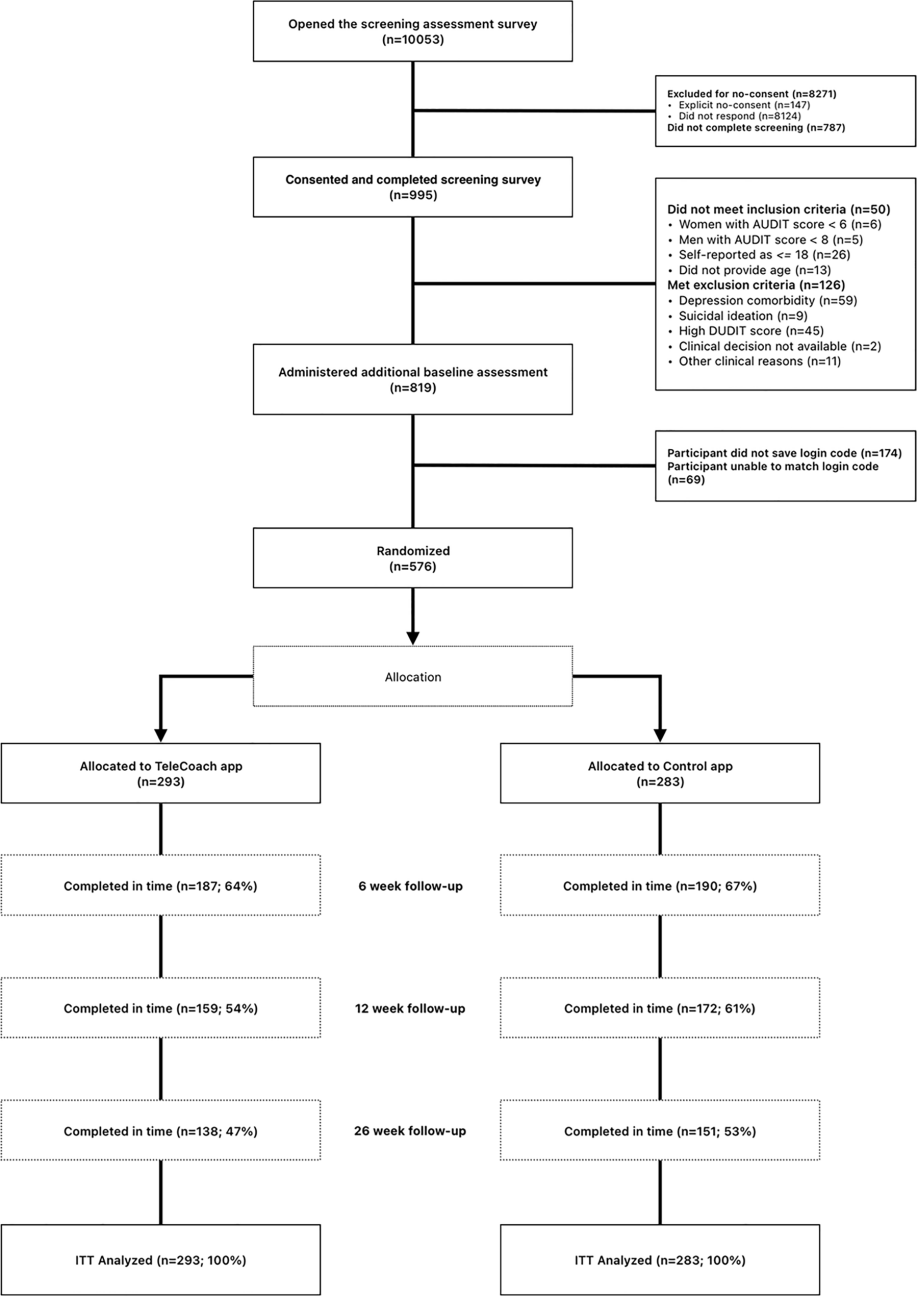
CONSORT diagram

Eligible participants were enrolled in the study between December 2018 and January 2020. Online recruitment attracted 10,053 visitors to the landing page for the screening survey, where 1929 (19.2%) initiated a survey response, 147 (7.6% of those initiating response) refused consent, 787 (40.8%) consented but did not complete the survey, and 995 (51.6%) consented and completed the survey. Of the survey completers, 50 (5%) did not fulfill inclusion criteria, and 126 (12.7%) fulfilled exclusion criteria, so a total of 819 (82.3%) were included in the study. Due to technical issues related to the automated login code, which required participants to save their login code and enter it into the app, an additional 243 participants (29.7%) were excluded. This procedure was meant to protect participant anonymity but led to some participants not saving their login code after baseline assessment, and others saving the code incorrectly; these cases had to be removed before randomization. In summary, 576 (70.3%) of the included participants were randomized and analyzed, with approximately half in each group (*n*_TeleCoach_ = 293; *n*_Control_ = 283). See Fig. 2 for an overview of the participant flow according to the CONSORT statement [78].

Regarding participant characteristics, women were the majority (67%), with somewhat more women in the control group (72% vs. 62%). Participants had a mean age of 47 years (SD = 11.21), most were married (72%), highly educated (59%), and employed (86%). Although over half of the participants reported at least three years of alcohol problems (65%), most had never sought help before (73%). See Table 1 for an overview of participant characteristics.

**Table 1:** Participant demographics

	Control (N = 283)	TeleCoach (N = 293)	Total (N = 576)
Women	72.08	61.77	66.84
Age: M (SD)	47.88 (11.28)	46.51 (11.12)	47.18(11.21)
Marital status			
Married	71.73	72.70	72.22
Widowed	8.48	7.51	7.99
Single	19.08	19.11	19.10
Other	0.71	0.68	0.69
Education - Junior high school			
Junior high school	4.24	4.10	4.17
High school	36.75	33.45	35.07
Undergraduate	48.06	48.46	48.26
Graduate	8.83	12.29	10.59
Other	2.12	1.71	1.91
Occupation			
Working	85.51	85.67	85.59
Sick leave	1.77	3.41	2.60
Seeking	2.47	3.07	2.78
Retired	8.13	6.14	7.12
Parental leave	1.06	0.68	0.87
Other	1.06	1.02	1.04
Duration of alcohol problems			
0–1 year	8.83	5.80	7.29
1–2 years	31.10	23.89	27.43
3–5 years	28.27	33.45	30.90
6–10 years	16.61	23.55	20.14
More than 10 years	14.13	12.97	13.54
No response	1.06	0.34	0.69
Help before			
Yes	27.92	24.57	26.22
No	72.08	74.4	73.26
No response	0	1.02	0.52

All numbers, except for age, are percentages

Baseline clinical characteristics (shown in Table 2) suggest participants in both groups were well above the threshold for problem drinking (i.e., group mean of 19.61 for AUDIT). The mean number of drinks in the past week was 27.61 (SD = 17.40). The mean number of drinks in a typical week was slightly lower at 23.19 (SD = 14.40). Participants reported drinking nearly five days in a typical week, about half of which were binge days. For each drinking session, the mean estimated blood alcohol concentration was 0.45‰ (SD = 0.34), and the mean peak was 1.07‰ (SD = 0.72). Notably, participants were also highly motivated, with a mean of 8.63 (SD = 1.73). Distributions for all variables concerning clinical characteristics are shown by group in Supplemental Fig. 1.

**Table 2:** Baseline clinical characteristics

	Control (N = 283)	TeleCoach (N = 293)	Total (N = 576)
AUDIT	19.29 (5.72)	19.92 (6.06)	19.61 (5.90)
Drinking quantity			
Past week (TLFB)	26.80 (16.72)	28.39 (18.02)	27.61 (17.40)
Typical week (DDQ)^†^	22.98 (14.00)	23.40 (14.79)	23.19 (14.40)
Drinking frequency (days/week)^‡^			
Typical frequency^†^	4.67 (1.82)	4.68 (1.74)	4.68 (1.78)
Typical binge frequency^†^	2.86 (2.05)	2.67 (1.99)	2.77 (2.02)
eBAC (‰)^‡^			
Typical Mean^†^	0.47 (0.36)	0.44 (0.32)	0.45 (0.34)
Typical Peak^†^	1.06 (0.73)	1.08 (0.71)	1.07 (0.72)
AUD Symptoms	5.94 (2.40)	6.49 (2.41)	6.22 (2.42)
PACS Total	14.14 (6.35)	14.82 (5.68)	14.48 (6.02)
AASE Total	20.25 (8.97)	20.02 (7.83)	20.13 (8.40)
Readiness Ruler	8.66 (1.76)	8.60 (1.71)	8.63 (1.73)
MADRS-S Total	14.04 (7.41)	15.26 (7.51)	14.66 (7.48)
GAD-7 Total	6.40 (4.92)	6.94 (5.00)	6.67 (4.97)

†DDQ data contained one invalid value in each group which was removed, resulting in fewer data points (*n* = 292 for TeleCoach; *n* = 282 for Controls; *n* = 574 total)

‡Drinking frequency and eBAC were derived from DDQ data

Note: AUDIT = Alcohol Use Disorders Identification Test; TLFB = Timeline Follow-back; DDQ = Daily Drinking Questionnaire; eBAC = estimated blood alcohol concentration; AUD = Alcohol use disorders; PACS = The Penn Alcohol Craving Scale; AASE = Alcohol Abstinence Self-efficacy Scale; MADRS-S = Montgomery-Åsberg Depression Rating Scale; GAD-7 = Generalized Anxiety Disorder 7-item scale. Unless otherwise noted, all values were presented in the *mean (standard deviation)* format

## Outcomes

### Attrition

Because recruitment occurred on an ongoing basis, follow-up times were determined relative to each participant’s time of enrollment into the study. Participants were reminded to complete their follow-up assessment by email. Some participants completed follow-up assessments outside the 30-day window at the 6-, 12-, and 26-week time points. At 6 weeks, 64% (*n* = 187) of participants in the intervention group completed the follow-up on time, compared to 67% (*n* = 190) in the control group. At 12 weeks, completion rates were 54% (*n* = 159) vs. 61% (*n* = 172), and at 26 weeks, 47% (*n* = 138) vs. 53% (*n* = 151), respectively. While the control group showed nominally higher on-time completion at each time point, these differences were not statistically significant (see Supplemental Table 2). The percentage of participants who returned for at least one valid follow-up was 74% in the intervention group and 81% in the control group. Outcome measurements collected outside the valid 30-day follow-up window were analyzed as missing values. See the CONSORT diagram in Fig. 2.

Three participants reported extremely high typical weekly alcohol consumption (i.e., more than 200 standard drinks; almost 10 times the sample mean) in response to the DDQ at baseline and 6-week follow-up. These participants were considered outliers and not included in any analyses.

### Primary outcomes

**Fig. 3:**
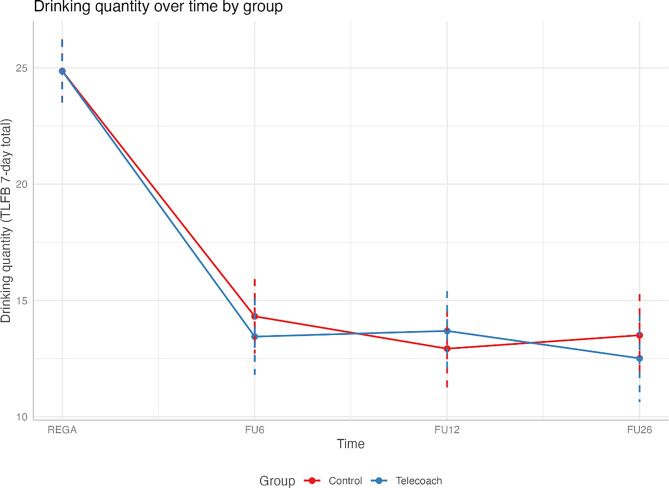
Visualization of analysis results for the primary hypothesis. This graph represents the estimated mean effects of the treatment and control groups

No significant differences occurred between the two groups at any follow-up. See Table 3 for detailed model estimates and Figure 3 for a visualization of results.

**Table 3:** Primary outcomes indicating change in past-week alcohol consumption compared to the control group at baseline

Variable	Estimate (B)	Std. Error	t	p	95% CI Lower	95% CI Upper
(Intercept)	16.10	2.18	7.37	0.00*	11.82	20.38
TeleCoach	0.65	0.99	0.65	0.51	−1.29	2.59
6-week follow-up	−10.57	0.83	−12.72	0.00*	−12.20	−8.94
12-week follow-up	−12.01	0.86	−13.89	0.00*	−13.70	−10.31
26-week follow-up	−11.44	0.91	−12.53	0.00*	−13.23	−9.65
Age	0.11	0.04	2.75	0.01*	0.03	0.18
Gender – Male	4.16	0.90	4.60	0.00*	2.39	5.92
Help before	0.74	0.96	0.78	0.44	−1.13	2.62
GAD 7	−0.08	0.10	−0.81	0.42	−0.28	0.12
MADRS	0.19	0.07	2.92	0.00*	0.06	0.32
TeleCoach * 6-week	−0.95	1.19	−0.80	0.42	−3.29	1.38
TeleCoach * 12-week	0.76	1.24	0.61	0.54	−1.68	3.19
TeleCoach * 26-week	−1.11	1.33	−0.83	0.41	−3.72	1.50

Note. Estimates were reported as contrasts; the intercept indicates the estimate for the control group, when the influence of time, covariates, and interactions were set to zero. Inclusion or exclusion of covariates did not alter results relevant to the primary hypothesis

**Table:** Random Effects: Primary Outcome

Variable	Variance	SD
Participant	63.22	7.95
Residual	8.23	3.00

### Secondary outcomes

**Within-group reduction.** Although not part of the primary between-group hypothesis, significant within-group reductions in alcohol consumption occurred over time in both groups. For the control group, estimates and effect sizes were as follows: *B* = −10.57, *SE* = 0.83, *95% CI* [−12.20, −8.94], *p* < 0.01, Cohen’s *d* = −1.28 at 6-week follow-up; *B* = −12.01, *SE* = 0.86, *95% CI* [−13.70, −10.31], *p* < 0.01, Cohen’s *d* = −1.45 at 12-week follow-up; and *B* = −11.44, *SE* = 0.91, *95% CI* [−13.23, −9.65], *p* < 0.01, Cohen’s *d* = −1.38 at 26-week follow-up. For the TeleCoach group, estimates and effect sizes were as follows: *B* = −11.52, *SE* = 1.45, 95% CI [−14.36, −8.68], *p* < 0.01, Cohen’s *d* = −1.39 at 6-week follow-up; *B* = −11.25, *SE* = 1.51, 95% CI [−14.21, −8.29], *p* < 0.01, Cohen’s *d* = −1.36 at 12-week follow-up; and *B* = −12.55, *SE* = 1.61, 95% CI [−15.71, −9.39], *p* < 0.01, Cohen’s *d* = −1.50 at 26-week follow-up. Time-invariant covariates, including older age (*B* = 0.11, *95% CI* [0.03, 0.18], *p* = 0.01), being male (*B* = 4.16, *95% CI* [2.39, 5.92], *p* < 0.01), and reporting more baseline symptoms of depression (*B* = 0.19, *95% CI* [0.06, 0.32], *p* < 0.01) were associated with higher drinking quantity over time in both groups. Zero-inflated negative binomial (ZINB) regression corroborated all the observed results, additionally indicated that all participants were likely to reduce alcohol consumption over time (*IRR* = 0.61, 0.57, 0.58 at 6-, 12-, and 26-week follow-up respectively; *p* < 0.001 for all follow-ups), as well as likely to achieve abstinence at follow-ups (*OR* = 35.49, 88.10, 45.34 at 6-, 12-, and 26-week follow-up respectively; *p* < 0.001 for all follow-ups) for all follow-ups; see Supplemental Table 3 for details).

**Fig. 4:**
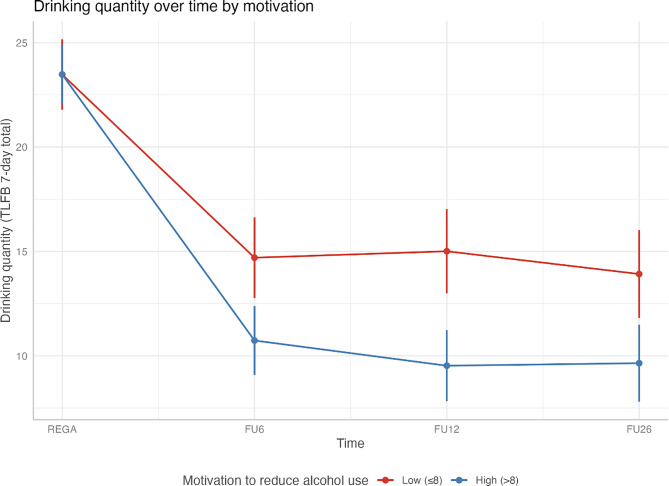
Visualization of reduction of weekly drinking quantity at four measure points in the total sample by high (>8) or low (<8) motivational level (scale 0–10). Note. motivation levels were dichotomized at ≤ 8 (low) and >8 (high) due to the highly skewed distribution of motivation scores in the sample. Most participants reported high motivation (median = 9, third quartile = 10 on the 0–10 scale), making the first quartile (≤8) a meaningful threshold to identify relatively less motivated participants

**Fig. 5:**
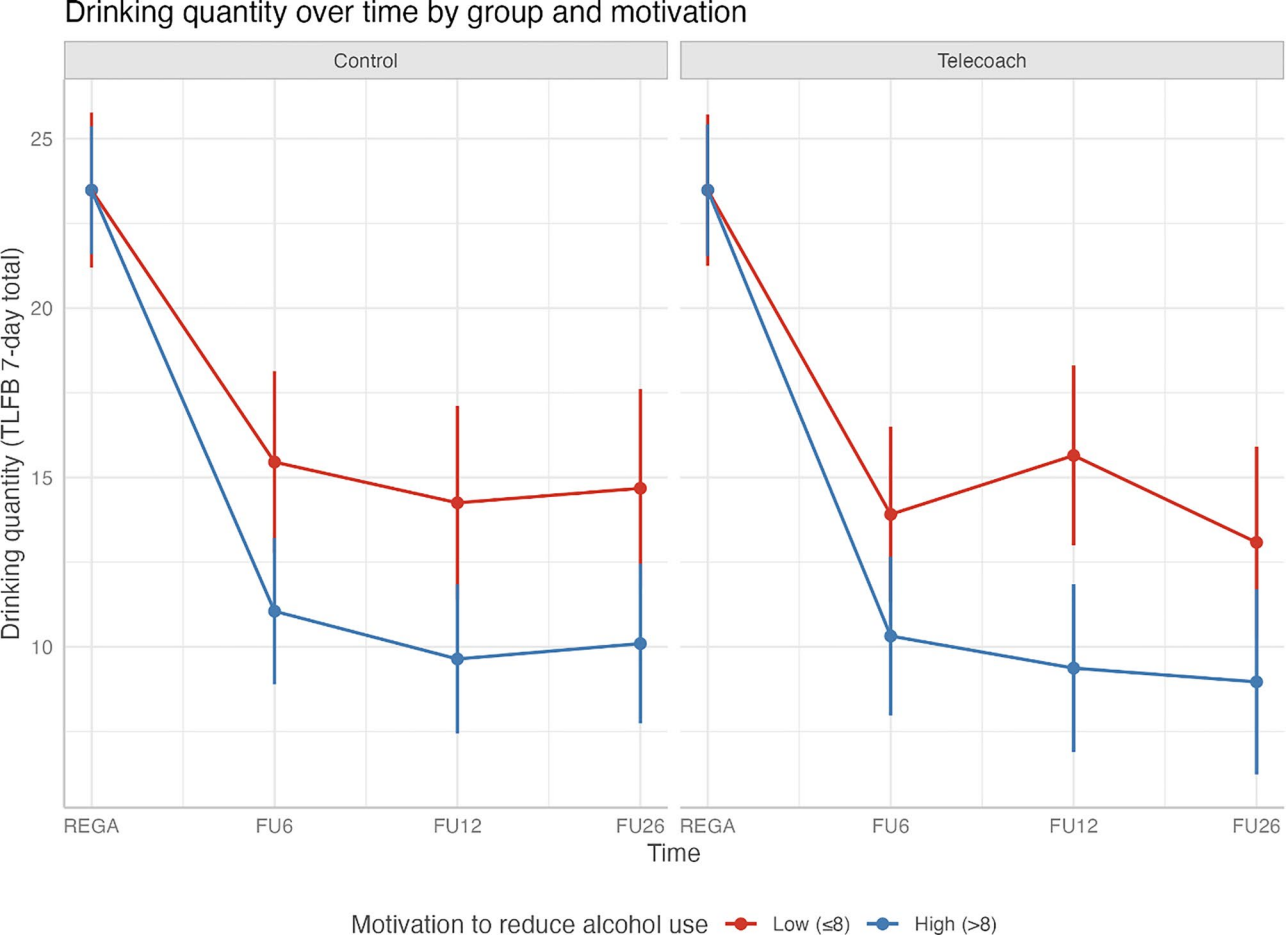
Visualization of reduction of weekly drinking quantity at four measure points by group and high (>8) or low (<8) motivational level (scale 0–10) after recoding motivation to high vs. low

**Role of Motivation.** In both groups, motivation was negatively associated with drinking at the 12- and 26-week follow-ups, such that higher motivation was associated with lower drinking levels (*B* = −0.88, *95% CI* [−1.55, −0.21], *p* = 0.01; *B* = −0.71, *95% CI* [−1.41, −0.01], *p* = 0.05), but not at 6-week follow-up (*B* = −0.62, *95% CI* [−1.77, 0.08], *p* = 0.07), with no differences between groups. See Table 4 for model estimates and Fig. 4 for a visualization of analysis results, where motivation was re-coded as high (>8) or low (≤8). Additional analyses per group showed no increased effects for more motivated individuals in the TeleCoach group. See Table 5 for model estimates, and Fig. 5 for visualization; see Supplemental Table 3 for ZINB models confirming all results.

**Table 4:** Secondary fixed effects indicating the overall effects of motivation over time

Variable	Estimate	Std. Error	t	p	95% CI Lower	95% CI Upper
(Intercept)	15.89	3.09	5.15	0.00*	9.84	21.94
6-week follow-up	−5.65	3.11	−1.82	0.07	−11.76	0.45
12-week follow-up	−4.06	3.01	−1.35	0.18	−9.96	1.83
26-week follow-up	−5.91	3.10	−1.91	0.06	−11.98	0.16
Motivation to reduce	0.03	0.28	0.11	0.92	−0.53	0.59
Age	0.11	0.04	2.87	0.00*	0.04	0.19
Gender – Male	4.16	0.90	4.64	0.00*	2.40	5.92
Help before	0.69	0.96	0.72	0.47	−1.18	2.57
GAD 7	−0.08	0.10	−0.77	0.44	−0.27	0.12
MADRS	0.19	0.07	2.96	0.00*	0.07	0.32
6-week follow-up * Motivation to reduce	−0.62	0.35	−1.77	0.08	−1.32	0.07
12-week follow-up * Motivation to reduce	−0.88	0.34	−2.57	0.01*	−1.55	−0.21
26-week follow-up * Motivation to reduce	−0.71	0.36	−2.00	0.05	−1.41	−0.01

Note. Estimates were reported as contrasts; the intercept indicates the estimate for the control group, when the influence of time, covariates, and interactions were set to zero

**Table:** Random Effects: Secondary Outcome 1

Variable	Variance	SD
Participant	63.79	7.99
Residual	8.15	3.00

**Table 5:** Secondary outcomes indicating the overall effects of motivation over time

Variable	Estimate	Std. Error	t	p	95% CI Lower	95% CI Upper
(Intercept)	12.12	3.94	3.08	0.00*	4.41	19.84
6-week follow-up	−1.54	4.59	−0.34	0.74	−10.54	7.46
12-week follow-up	−3.21	4.36	−0.74	0.46	−11.75	5.33
26-week follow-up	−3.61	4.47	−0.81	0.42	−12.38	5.15
TeleCoach	8.30	4.99	1.66	0.10	−1.48	18.08
Motivation to reduce	0.44	0.40	1.09	0.28	−0.35	1.22
Age	0.11	0.04	2.80	0.01*	0.03	0.19
Gender – Male	4.19	0.91	4.61	0.00*	2.40	5.97
Help before	0.65	0.96	0.67	0.50	−1.24	2.54
GAD 7	−0.08	0.10	−0.79	0.43	−0.28	0.12
MADRS	0.20	0.07	3.00	0.00*	0.07	0.33
6-week * TeleCoach	−8.08	6.29	−1.29	0.20	−20.40	4.24
12-week * TeleCoach	−2.58	6.06	−0.42	0.67	−14.46	9.31
26-week * TeleCoach	−4.82	6.24	−0.77	0.44	−17.05	7.40
6-week follow-up * Motivation to reduce	−1.04	0.52	−2.02	0.04*	−2.05	−0.03
12-week follow-up * Motivation to reduce	−1.01	0.49	−2.07	0.04*	−1.97	−0.05
26-week follow-up * Motivation to reduce	−0.91	0.51	−1.80	0.07	−1.90	0.08
TeleCoach * Motivation to reduce	−0.89	0.57	−1.56	0.12	−2.00	0.23
6-week follow-up * TeleCoach * Motivation to reduce	0.81	0.71	1.14	0.26	−0.59	2.21
12-week follow-up * TeleCoach * Motivation to reduce	0.36	0.69	0.53	0.60	−0.99	1.72
26-week follow-up * TeleCoach * Motivation to reduce	0.41	0.72	0.57	0.57	−0.99	1.81

Note. Estimates were reported as contrasts; the intercept indicates the estimate for the control group, when the influence of time, covariates, and interactions were set to zero

**Table:** Random Effects: Secondary Outcome 2

Variable	Variance	SD
Participant	64.34	8.02
Residual	8.18	3.00

Note. Estimates were reported as contrasts; the intercept indicates the estimate for the control group, when the influence of time, covariates, and interactions were set to zero

**Fig. 6:**
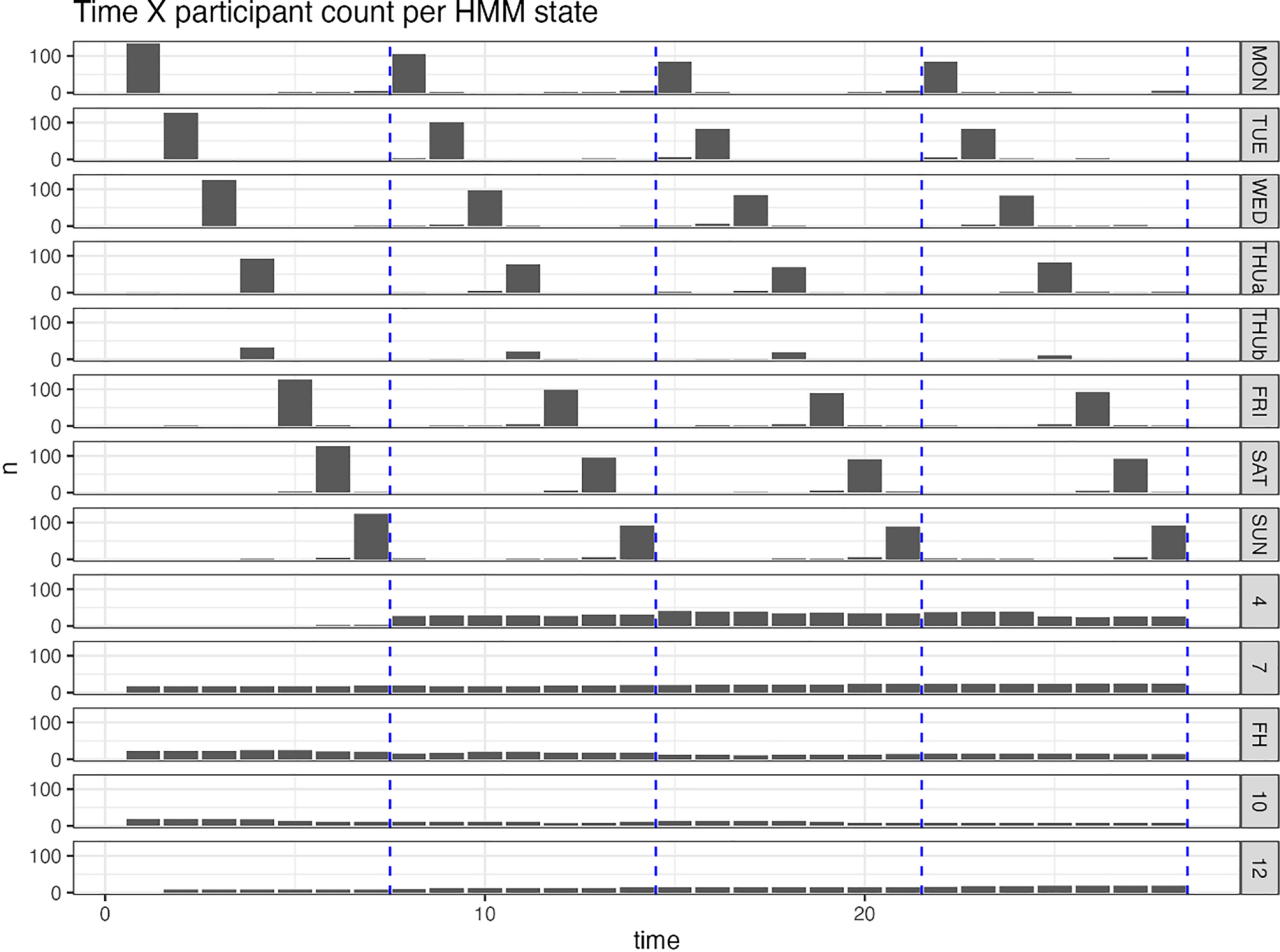
Participant count per state over time

**Frequent-Heavy Subgroup.** The Hidden Markov analysis identified seven day-of-the-week states and a frequent-heavy (FH) subgroup in a 13-state model (see Table 6 and Fig. 6). However, the FH subgroup was small (*n* = 38), and confirmatory analysis did not reveal significant differences in heavy (binge) drinking days between the intervention and the control group at any follow-ups. See Supplemental Figure 2 for supplementary visualizations.

**Table 6:** Response parameters for the 13-state hidden Markov Model

State	Abstinent	Moderate	Binge	N
MON	0.83	0.12	0.05	154
TUE	0.79	0.17	0.04	146
WED	0.59	0.29	0.12	146
THUa	0.92	0.00	0.08	131
THUb	0.01	0.99	0.00	64
FRI	0.08	0.29	0.63	147
SAT	0.07	0.26	0.67	146
SUN	0.68	0.24	0.08	144
4	1.00	0.00	0.00	58
7	0.33	0.64	0.03	29
**FH**	**0.09**	**0.05**	**0.86**	**38**
10	0.00	0.82	0.18	27
12	0.79	0.02	0.19	18

Note. FH = Frequent-Heavy excessive drinking pattern

**Excessive Drinking.** Regarding excessive drinking, in the total sample at baseline, 86% of the participants were drinking at excessive levels in a typical week based on the original gender-specific definition of excessive drinking. The proportion of participants with excessive drinking in the total sample dropped to 30% by 6 weeks (*X*^*2*^(1, *n* = 325) = 18.75, *p* < 0.001), and to 26 and 23% at 12- and 26-week follow-ups, respectively, with no differences between groups, although between-group analyses nominally favored the intervention group at 12-week follow-up across per-protocol and imputed datasets, regardless of the measurement (i.e., DDQ and TLFB) or prior/revised standards for excessive drinking. See Supplemental Table 1 for details.

No harm or unintended effects were observed by the clinical psychologists (authors AHB and OM) or reported by any participants.

### Usage data

For the TeleCoach app, the two units in the “Intake and hazardous drinking” component were the most visited (see Fig. 1 for an overview of components and units). On average, participants visited the “Register intake” unit 3 times throughout the study period (*SD* = 3.96). The mean number of visits per user was 2.3 for the risk education unit (i.e., “Hazardous drinking”; *SD* = 1.79). The third most-visited unit was the one designed to deliver personalized skills training recommendations based on the AASE; the mean visits per user for this unit was 1.85 (*SD* = 1.36). The least visited unit was “Confident body language,” where the mean visit per user was 0.56 (*SD =* 0.83). More details are shown in Supplemental Fig. 3. For the control app, the unit dedicated to risk education about hazardous drinking was the most visited per user (*M* = 3.53, *SD* = 3.23), followed by gender-specific risk education units (*M*_Men_ = 2.30, *SD* = 2.28; *M*_Women_ = 2.34, *SD* = 1.71). The unit about early signs of harmful drinking was the least visited, but the nominal difference was small (*M* = 2.03, *SD* = 1.54). More details are shown in Supplemental Fig. 4.

## Discussion

This randomized controlled trial aimed to evaluate the effectiveness of an unguided, automated skills training app for problem drinking, with the primary hypothesis that participants in the intervention app group would drink less at all follow-up timepoints compared to the control group. The secondary hypotheses concerned an expected amplified intervention effect for highly motivated participants, for participants with a frequent-heavy pattern of drinking, and excessive drinkers. None of the hypotheses were supported, but participants in both groups reduced their drinking over time. Participants in both groups reported reducing their drinking by approximately 11 drinks per week over time, with no differences between the intervention and the control apps at the 6-, 12-, and 26-week follow-ups. Participants with higher motivation in both groups tended to report lower drinking at 12- and 26-week follow-ups. A small frequent-heavy subgroup was identified in the sample, but participants with a frequent-heavy drinking pattern did not differ between app groups regarding the number of reduced drinking days over time. The proportion of participants with excessive drinking declined markedly over time in both groups, with no difference between the intervention and the control groups. In summary, no between-group differences occurred, but multiple significant within-group reductions in drinking levels across time were found in both groups.

Several factors may explain the lack of significant between-group differences. Participant characteristics in this study highlight essential differences from previous research and may offer alternative explanations for these findings. Specifically, the study recruitment process and inclusion criteria attracted a group of very highly motivated participants—the mode value for readiness to change was 10 out of 10 in both groups (47%; see Supplemental Fig. 1). The younger participants in the previous two TeleCoach trials had lower problem severity, with AUDIT scores ranging from 11 - 14, and motivation levels of 4 to 5 [24, 25]. In this trial, the mean AUDIT score was 20, corresponding to probable dependence, similar to the problem severity reported in a sample of adults diagnosed with alcohol use disorder who were recruited via the internet to a randomized trial on internet-delivered 13-week treatment [79]. The participants’ high motivation to change and high severity of alcohol use problems, combined with their long-standing problem history and lack of previous help-seeking characteristics may have put them at a tipping point where they were especially receptive to an app-based intervention, even one with minimal content.

Our findings thus suggest that, among highly motivated internet help-seekers with a high level of pre-existing problem drinking, the enhanced skills training content did not produce additional benefit beyond basic risk education. However, without a passive control group, it remains unclear whether the reductions observed in both groups reflect an effect of either app or other factors. The exceptionally high baseline motivation in our sample may imply that most participants had already progressed beyond the stage where motivational components of interventions would be most beneficial. Different components of alcohol interventions are designed to address distinct stages in the change process. Personalized feedback, a common component of brief alcohol interventions, typically aims to increase motivation by enhancing problem awareness and highlighting discrepancies between current behavior and health-related goals [80, 81], while skills training components, in contrast, are designed to provide tools for implementing change and coping with potential relapse scenarios once the motivation is established. While higher baseline motivation in our study predicted lower alcohol consumption at later follow-ups, these already highly motivated participants may not have benefitted from the intervention app’s enhanced features. The ceiling effect in baseline motivation may have masked any potential impact of personalized feedback in further enhancing participants’ readiness to change. At the same time, the other main component of the intervention app, skills training, did not seem to make a detectable difference among these highly motivated participants. One possibility is that skills training offered in the intervention app might be more beneficial at moderate levels of motivation—when individuals are somewhat ready to change but need concrete strategies to implement their intentions and cope with any drawbacks associated with fewer drinking. At very high levels of motivation, as seen in our sample, participants may independently develop and implement their change strategies, making formal skills training less valuable. Therefore, motivation could be a shared mechanism of change across both groups.

Nevertheless, the lack of a significant between-group effect on alcohol consumption in this study is consistent with other recently published studies that also compared enhanced apps to education-focused app content. Oldham et al. [20] compared Drink Less to a control condition similar to our risk-education-based control app—in a highly motivated population with elevated baseline AUDIT scores (mean 21.9). The primary analysis in their study showed a non-significant reduction of 0.98 units, with a significant two-unit reduction in favor of the app emerging only in sensitivity analyses using multiple imputations. and Cunningham et al. [21] also compared a full intervention app against an education-only control, finding a modest reduction of about 2.6 drinks per week. However, motivation was not reported in these studies as a possible moderator. The considerable reduction in alcohol use over time, along with the lack of significant between-group differences, could be attributed to regression towards the mean or spontaneous recovery [82]. However, this explanation is inconsistent with the earlier finding that access to the intervention app, TeleCoach, was associated with less alcohol use compared to the assessment-only control condition [25]. Since this study did not include a passive control, it is not possible to disentangle the effects of having access to an app or not.

Usage statistics in this study indicated that participants in both conditions seemed adequately engaged with the app. The mean number of visits per unit per user indicated that all except the “Confident body language” unit in the intervention app were visited at least once. In contrast, participants in the control app visited all units at least twice. The body language unit in the intervention app was the only unit not connected to the personalized skill training recommendation algorithm in the “Risk situations” unit (see Fig. 1). This can be interpreted as indirect evidence that most participants engaged meaningfully with the personalized recommendation design. In the control app, participants were somewhat more engaged with individual units, where the minimum number of visits per unit per user was above two. Given the high motivation among participants in both groups, it is possible that control group participants returned to the app seeking further intervention content, although no additional material was provided in the control app.

## Strengths and limitations

The major strength of this study was the low threshold for participation. Of the 995 consenting participants who completed the screening survey, only 5% were excluded due to not meeting inclusion criteria for problem drinking, while 12.7% met exclusion criteria, allowing for 82.3% to be included. Most of the participants reported more than 3 years of alcohol problems, but most had never sought help prior to this study. These findings provide evidence that a low-threshold, app-based intervention can reach a broad and diverse population of adult internet help-seekers who might not otherwise seek treatment for problem drinking. A second significant strength is that the intervention app has been rebuilt to ensure it remains accessible for further evaluation and/or public access, with an open-source approach to preserve the intervention app and analysis code for future reference and use. Double-blinding is a further methodological strength of this study. Interestingly, however, several participants contacted the researchers to indicate that they realized they were in the control group due to the brevity of the control app. Future studies could strengthen and verify the credibility of active control apps.

This study was somewhat limited by its two-armed design. The active control condition limited the potential confounding effects of receiving any treatment. Still, the lack of an additional waitlist or assessment-only control group precluded the possibility of addressing other potential biases in the study, such as regression to the mean. However, a no-treatment waitlist group design could have raised ethical concerns since it is known that any digital intervention could have *some* positive effects [18, 83], and with other unvalidated apps available on the market, preventing already highly motivated participants from accessing any app at all for the waitlist period could be questionable. We suggest that the improvement over time seen in both conditions is unlikely to be solely attributable to regression to the mean, given the medium-to-large effect sizes that were sustained over the entire follow-up period, for up to 26 weeks. Indeed, the within-group effect sizes in the current study were close to the effect sizes of an earlier trial of a more intensive, 12-week, CBT-based unguided digital intervention program [79]. Notably, the 2020 trial included a waitlist control group, which did not show similar levels of improvement over time.

Another study limitation concerned the recruitment process. Pre-screening participants based on inclusion/exclusion criteria before randomization and treatment access is a common practice in clinical trials. However, the screening process required potential participants to put in substantial efforts to access an intervention app. In contrast, most commercially available apps that participants are familiar with are often optimized for effortless and instantaneous availability. Consequently, it is likely that only the most motivated individuals will complete the sign-up process when entering a research trial, limiting the observed primary outcomes to this group of highly motivated individuals. Similarly, the observed 10% approximate conversion rate from clicking on the online advertisement to completing the process of signing up for app access may not generalize to a situation where the app would be publicly accessible without pre-screening questionnaires.

Other common limitations were also present in this study. Participant attrition occurred in both conditions, though the attrition rate was moderate, and most participants completed at least one follow-up. Relying on emails to contact participants for follow-up surveys might have contributed to attrition, but the evidence for the relative efficacy of other reminder strategies is unclear [84–86]. The generalizability of this study was also limited by the sampled population. Participants were all drawn from Sweden, a western, educated, industrialized, rich, and democratic society, so the findings might not generalize to developing countries.

## Conclusions

In conclusion, this trial provides evidence that app-based interventions can serve as a scalable approach to reach individuals with problem drinking—particularly motivated adults with high problem severity actively seeking help online. Most participants had experienced alcohol-related problems for at least three years, yet had not previously sought professional support, highlighting the potential of digital tools to engage this important but often overlooked group. The results contribute tentative evidence that low-threshold and low-intensity app-based interventions can robustly support individuals highly motivated to change their drinking habits in a healthy direction. Future studies should build on a recognition that less motivated help-seekers may have treatment needs that significantly differ from the treatment needs of more motivated individuals. Studies will require innovative approaches to identify and engage less motivated individuals who might benefit more from enhanced app content and interactivity.

## Electronic supplementary material

Below is the link to the electronic supplementary material.

Supplementary material 1

## Data Availability

The deidentified raw data supporting the conclusions of this article, along with all analytic code, will be made available upon reasonable request.

## References

[1] Degenhardt L, Charlson F, Ferrari A, Santomauro D, Erskine H, Mantilla-Herrara A, et al. The global burden of disease attributable to alcohol and drug use in 195 countries and territories, 1990-2016. A systematic analysis for the global burden of disease study 2016. Lancet Psychiatry. 2018, Dec;5(12):987–1012. 10.1016/S2215-0366(18)30337-7. 30392731 10.1016/S2215-0366(18)30337-7 PMC6251968

[2] Manthey J, Hassan SA, Carr S, Kilian C, Kuitunen-Paul S, Rehm J. What are the economic costs to society attributable to alcohol use? A systematic review and modelling study. PharmacoEconomics. 2021, Jul;39(7):809–22. 10.1007/s40273-021-01031-8. 33970445 10.1007/s40273-021-01031-8 PMC8200347

[3] Rehm J, Mathers C, Popova S, Thavorncharoensap M, Teerawattananon Y, Patra J. Global burden of disease and injury and economic cost attributable to alcohol use and alcohol-use disorders. Lancet. 2009, Jun;373(9682):2223–33. 10.1016/S0140-6736(09)60746-7. 19560604 10.1016/S0140-6736(09)60746-7

[4] American Psychiatric Association. Diagnostic and statistical manual of mental disorders: dSM-5-TR, editor. Text revision. Fifth. Washington, DC: American Psychiatric Association Publishing; 2022. p. 1 p.

[5] Tuithof M, Ten Have M, Van Den Brink W, Vollebergh W, De Graaf R. The relationship between excessive alcohol consumption and alcohol use disorders according to DSM-IV and DSM-5. Alcohol Clin Exp Res. 2014, Jan;38(1):249–56. 10.1111/acer.12248. 24033529 10.1111/acer.12248

[6] Finn SW, Mejldal A, Nielsen AS. Perceived barriers to seeking treatment for alcohol use disorders among the general Danish population - a cross sectional study on the role of severity of alcohol use and gender. Arch Public Health. 2023, Apr, 23;81(1):65. 10.1186/s13690-023-01085-4. 37087483 10.1186/s13690-023-01085-4 PMC10122805

[7] Grigg J, Manning V, Cheetham A, Youssef G, Hall K, Baker AL, et al. A latent class analysis of perceived barriers to help-seeking among people with alcohol use problems presenting for telephone-delivered treatment. Alcohol Alcohol. 2023, Jan, 9;58(1):68–75. 10.1093/alcalc/agac063. 10.1093/alcalc/agac063 PMC9830485 36448844

[8] Probst C, Manthey J, Martinez A, Rehm J. Alcohol use disorder severity and reported reasons not to seek treatment: a cross-sectional study in European primary care practices. Subst Abuse Treat Prev Policy. 2015, Dec;10(1):32. 10.1186/s13011-015-0028-z. 26264215 10.1186/s13011-015-0028-z PMC4534056

[9] Schuler MS, Puttaiah S, Mojtabai R, Crum RM. Perceived barriers to treatment for alcohol problems: a latent class analysis. PS. 2015, Nov;66(11):1221–28. 10.1176/appi.ps.201400160. 10.1176/appi.ps.201400160 PMC4630073 26234326

[10] Mekonen T, Chan GCK, Connor J, Hall W, Hides L, Leung J. Treatment rates for alcohol use disorders: a systematic review and meta-analysis. Addiction. 2021, Oct;116(10):2617–34. 10.1111/add.15357. 33245581 10.1111/add.15357

[11] Roerecke M, Gual A, Rehm J. Reduction of alcohol consumption and subsequent mortality in alcohol use disorders: systematic review and meta-analyses. J Clin Psychiatry. 2013, Dec, 15;74(12):e1181–9. 10.4088/JCP.13r08379. 10.4088/JCP.13r08379 24434106

[12] Schueller SM, Torous J. Scaling evidence-based treatments through digital mental health. Am Psychol. 2020, Nov;75(8):1093–104. 10.1037/amp0000654. 33252947 10.1037/amp0000654 PMC7709142

[13] Gomes M, Murray E, Raftery J. Economic evaluation of digital health interventions: methodological issues and recommendations for practice. PharmacoEconomics. 2022, Apr;40(4):367–78. 10.1007/s40273-022-01130-0. 35132606 10.1007/s40273-022-01130-0 PMC8821841

[14] Fu Z, Burger H, Arjadi R, Bockting CLH. Effectiveness of digital psychological interventions for mental health problems in low-income and middle-income countries: a systematic review and meta-analysis. Lancet Psychiatry. 2020, Oct;7(10):851–64. 10.1016/S2215-0366(20)30256-X. 32866459 10.1016/S2215-0366(20)30256-X PMC7455253

[15] Shanahan M, Bahia K. The state of mobile internet connectivity 2023 [Internet]. 2023, Oct. https://www.gsma.com/r/wp-content/uploads/2023/10/The-State-of-Mobile-Internet-Connectivity-Report-2023.pdf. cited 2024 Apr 30]. Report No. Available from]. London, United Kingdom: GSMA.

[16] Silver L. Smartphone ownership is growing rapidly around the world, but not always equally [internet]. 2019, Feb. https://www.pewresearch.org/global/2019/02/05/smartphone-ownership-is-growing-rapidly-around-the-world-but-not-always-equally/. cited 2024 Apr 29]. Report No. Available from. Pew Research Center.

[17] Lattie EG, Stiles-Shields C, Graham AK. An overview of and recommendations for more accessible digital mental health services. Nat Rev Psychol. 2022, Jan, 26;1(2):87–100. 10.1038/s44159-021-00003-1. 38515434 10.1038/s44159-021-00003-1 PMC10956902

[18] Riper H, Hoogendoorn A, Cuijpers P, Karyotaki E, Boumparis N, Mira A, et al. Effectiveness and treatment moderators of internet interventions for adult problem drinking: an individual patient data meta-analysis of 19 randomised controlled trials. PLoS Med. 2018, Dec, 18;15(12):e1002714. 10.1371/journal.pmed.1002714. Degenhardt, L, editor. 10.1371/journal.pmed.1002714 PMC6298657 30562347

[19] Sohi I, Shield KD, Rehm J, Monteiro M. Digital interventions for reducing alcohol use in general populations: an updated systematic review and meta-analysis. Alcohol: Clin Exp Res. 2023, Oct;47(10):1813–32. 10.1111/acer.15175. 10.1111/acer.15175 37864535

[20] Oldham M, Beard E, Loebenberg G, Dinu L, Angus C, Burton R, et al. Effectiveness of a smartphone app (Drink Less) versus usual digital care for reducing alcohol consumption among increasing-and-higher-risk adult drinkers in the Uk: a two-arm, parallel-group, double-blind, randomised controlled trial. eClinicalmedicine. 2024, Apr;70:102534. 10.1016/j.eclinm.2024.102534. 38685934 10.1016/j.eclinm.2024.102534 PMC11056393

[21] Cunningham JA, Godinho A, Schell C, Studer J, Wardell JD, Garnett C, et al. Randomized controlled trial of a smartphone app designed to reduce unhealthy alcohol consumption. Internet Interventions. 2024, Jun;36:100747. 10.1016/j.invent.2024.100747. 38812955 10.1016/j.invent.2024.100747 PMC11133919

[22] Farren C, Farrell A, Hagerty A, McHugh C. A 6-month randomized trial of a smartphone application, UControlDrink, in aiding recovery in alcohol use disorder. Eur Addict Res. 2022;28(2):122–33. 10.1159/000519945. 34802002 10.1159/000519945

[23] Gustafson DH, McTavish FM, Chih MY, Atwood AK, Johnson RA, Boyle MG, et al. A smartphone application to support recovery from alcoholism: a randomized clinical trial. JAMA Psychiatry. 2014, May;71(5):566–72. 10.1001/jamapsychiatry.2013.4642 PubMed PMID: 24671165; PubMed Central PMCID: PMC4016167. 24671165 10.1001/jamapsychiatry.2013.4642 PMC4016167

[24] Berman AH, Andersson C, Gajecki M, Rosendahl I, Sinadinovic K, Blankers M. Smartphone apps targeting hazardous drinking patterns among university students show differential subgroup effects over 20 weeks: results from a randomized, controlled trial. JCM. 2019, Oct, 28;8(11):1807. 10.3390/jcm8111807. 31661868 10.3390/jcm8111807 PMC6912621

[25] Gajecki M, Andersson C, Rosendahl I, Sinadinovic K, Fredriksson M, Berman AH. Skills training via smartphone app for university students with excessive alcohol consumption: a randomized controlled trial. IntJ Behav Med. 2017, Oct;24(5):778–88. 10.1007/s12529-016-9629-9. 28224445 10.1007/s12529-016-9629-9 PMC5608866

[26] Andersson C, Gajecki M, Öjehagen A, Berman AH. Automated telephone interventions for problematic alcohol use in clinical and population samples: a randomized controlled trial. BMC Res Notes. 2017, Dec;10(1):624. 10.1186/s13104-017-2955-4. 29183357 10.1186/s13104-017-2955-4 PMC5704400

[27] The Swedish National Board of Health and Welfare. Kunskapsunderlag - insatser vid riskbruk av alkohol [Knowledge base - interventions for risky use of alcohol]. Report No. 2023. Stockholm: Socialstyrelsen [The Swedish National Board of Health and Welfare].

[28] Berman AH, Molander O, Tahir M, Törnblom P, Gajecki M, Sinadinovic K, et al. Reducing risky alcohol use via smartphone app skills training among adult internet help-seekers: a randomized pilot trial. Front. Psychiatry. 2020, May;27(11):434. 10.3389/fpsyt.2020.00434. 10.3389/fpsyt.2020.00434 PMC7267061 32536880

[29] Sinadinovic K, Wennberg P, Berman AH. Short-term changes in substance use among problematic alcohol and drug users from a general population sample. IJADR. 2014, Dec, 11;3(4):277–87. 10.7895/ijadr.v3i4.186.

[30] Saunders JB, Aasland OG, Babor TF, De La Fuente JR, Grant M. Development of the alcohol use disorders Identification Test (AUDIT): WHO collaborative project on early detection of persons with harmful alcohol consumption‑II. Addiction. 1993, Jun;88(6):791–804. 10.1111/j.1360-0443.1993.tb02093.x. 8329970 10.1111/j.1360-0443.1993.tb02093.x

[31] Bergman H. Alcohol use among swedes and a psychometric evaluation of the alcohol use disorders identification test. Alcohol Alcohol. 2002, May, 1;37(3):245–51. 10.1093/alcalc/37.3.245. 12003912 10.1093/alcalc/37.3.245

[32] Montgomery SA, Åsberg M. A new depression scale designed to be sensitive to change. Br J Psychiatry. 1979, Apr;134(4):382–89. 10.1192/bjp.134.4.382. 444788 10.1192/bjp.134.4.382

[33] Berman AH, Bergman H, Palmstierna T, Schlyter F. Evaluation of the drug use disorders Identification Test (DUDIT) in criminal justice and detoxification settings and in a Swedish population sample. Eur Addict Res. 2005;11(1):22–31. 10.1159/000081413. 15608468 10.1159/000081413

[34] Voluse AC, Gioia CJ, Sobell LC, Dum M, Sobell MB, Simco ER. Psychometric properties of the drug use disorders Identification Test (DUDIT) with substance abusers in outpatient and residential treatment. Addictive Behaviors. 2012, Jan;37(1):36–41. 10.1016/j.addbeh.2011.07.030. 21937169 10.1016/j.addbeh.2011.07.030

[35] Sobell LC, Sobell MB. Timeline follow-back: a technique for assessing self-reported alcohol consumption. In: Litten RZ, Allen JP, editors. Measuring alcohol consumption [internet]. Totowa, NJ: Humana Press; 1992. p. 41–72. Available from: cited 2024 Apr 8. 10.1007/978-1-4612-0357-5_3. http://link.springer.com/10.1007/978-1-4612-0357-5_3.

[36] Collins RL, Parks GA, Marlatt GA. Social determinants of alcohol consumption: the effects of social interaction and model status on the self-administration of alcohol. J Consult Clin Psych. 1985, Apr;53(2):189–200. 10.1037/0022-006X.53.2.189. 10.1037//0022-006x.53.2.189 3998247

[37] Piumatti G, Aresi G, Marta E. A psychometric analysis of the daily drinking questionnaire in a nationally representative sample of young adults from a Mediterranean drinking culture. J Ethnicity Subst Abuse. 2023, Feb, 1;22(1):171–88. 10.1080/15332640.2021.1918600. 10.1080/15332640.2021.1918600 34003733

[38] Carey KB, Carey MP, Maisto SA, Henson JM. Temporal stability of the timeline followback interview for alcohol and drug use with psychiatric outpatients. J Stud Alcohol. 2004, Nov;65(6):774–81. 10.15288/jsa.2004.65.774. 15700516 10.15288/jsa.2004.65.774 PMC2424021

[39] Roy M, Dum M, Sobell LC, Sobell MB, Simco ER, Manor H, et al. Comparison of the quick drinking screen and the alcohol Timeline Followback with outpatient alcohol abusers. Subst Use & Misuse. 2008, Dec, 16;43(14):2116–23. 10.1080/10826080802347586. 10.1080/10826080802347586 18825590

[40] Sobell LC, Brown J, Leo GI, Sobell MB. The reliability of the alcohol Timeline Followback when administered by telephone and by computer. Drug Alcohol Depen. 1996, Sep;42(1):49–54. 10.1016/0376-8716(96)01263-X. 10.1016/0376-8716(96)01263-x 8889403

[41] Hareskov Jensen N, Vallentin-Holbech L, Dash GF, Feldstein Ewing SW, Rømer Thomsen. Validity of an online, self-administered Timeline Followback for alcohol use with adolescents. Front. Psychiatry. 2023, Nov;30(14):1221487. 10.3389/fpsyt.2023.1221487. 10.3389/fpsyt.2023.1221487 PMC10720705 38098631

[42] Pedersen ER, Grow J, Duncan S, Neighbors C, Larimer ME. Concurrent validity of an online version of the Timeline Followback assessment. Phychol Addictive Behaviors. 2012, Sep;26(3):672–77. 10.1037/a0027945. 10.1037/a0027945 PMC3714014 22486334

[43] Hjorthøj CR, Hjorthøj AR, Nordentoft M. Validity of Timeline follow-back for self-reported use of cannabis and other illicit substances - systematic review and meta-analysis. Addictive Behaviors. 2012, Mar;37(3):225–33. 10.1016/j.addbeh.2011.11.025. 22143002 10.1016/j.addbeh.2011.11.025

[44] Hoeppner BB, Stout RL, Jackson KM, Barnett NP. How good is fine-grained Timeline follow-back data? Comparing 30-day TLFB and repeated 7-day TLFB alcohol consumption reports on the person and daily level. Addictive Behaviors. 2010, Dec;35(12):1138–43. 10.1016/j.addbeh.2010.08.013. 20822852 10.1016/j.addbeh.2010.08.013 PMC2942970

[45] Alcohol Research: current Reviews editorial S. Drinking patterns and their definitions. ARCR. 2018;39(1):17. 10.35946/arcr.v39.1.03. 10.35946/arcr.v39.1.03 PMC6104961 30557143

[46] Gajecki M, Berman AH, Sinadinovic K, Rosendahl I, Andersson C. Mobile phone brief intervention applications for risky alcohol use among university students: a randomized controlled study. Addict Sci Clin Pract. 2014, Dec;9(1):11. 10.1186/1940-0640-9-11. 24985342 10.1186/1940-0640-9-11 PMC4091647

[47] Miller WR, Rollnick S. Motivational interviewing: helping people change. 3rd. New York, NY: Guilford Press; 2013. p. 482 p. (Applications of motivational interviewing.

[48] Gaume J, Bertholet N, Daeppen JB. Readiness to change predicts drinking: findings from 12-month follow-up of alcohol use disorder outpatients. Alcohol Alcohol. 2017, Jan;52(1):65–71. 10.1093/alcalc/agw047. 27469491 10.1093/alcalc/agw047

[49] Harris TR, Walters ST, Leahy MM. Readiness to change among a group of heavy-drinking college students: correlates of readiness and a comparison of measures. J Am Coll Health. 2008, Nov;57(3):325–30. 10.3200/JACH.57.3.325-330. 18980889 10.3200/JACH.57.3.325-330

[50] Heather N, Smailes D, Cassidy P. Development of a readiness Ruler for use with alcohol brief interventions. Drug Alcohol Depen. 2008, Dec;98(3):235–40. 10.1016/j.drugalcdep.2008.06.005. 10.1016/j.drugalcdep.2008.06.005 18639393

[51] St-Hilaire A, Axelrod K, Geller J, Mazanek Antunes J, Steiger H. A readiness Ruler for assessing motivation to change in people with eating disorders. Euro Eat Disord Rev. 2017, Sep;25(5):417–22. 10.1002/erv.2533. 10.1002/erv.2533 28695662

[52] Chen Y, Wills L, Wise H, Erford BT, Yao R. Psychometric synthesis of the alcohol use disorders Identification Test (AUDIT). Jour Couns Develop. 2024, Oct;102(4):406–14. 10.1002/jcad.12531.

[53] Svanborg P, Åsberg M. A comparison between the beck depression inventory (BDI) and the self-rating version of the Montgomery Åsberg depression rating scale (MADRS). J Educ Chang Affective Disord. 2001, May;64(2–3):203–16. 10.1016/S0165-0327(00)00242-1. 10.1016/s0165-0327(00)00242-1 11313087

[54] Müller M, Himmerich H, Kienzle B, Szegedi A. Differentiating moderate and severe depression using the Montgomery-Åsberg depression rating scale (MADRS). J Educ Chang Affective Disord. 2003, Dec;77(3):255–60. 10.1016/S0165-0327(02)00120-9. 10.1016/s0165-0327(02)00120-9 14612225

[55] Fantino B, Moore N. The self-reported Montgomery-Åsberg depression rating scale is a useful evaluative tool in major depressive disorder. BMC Psychiatry. 2009, Dec;9(1):26. 10.1186/1471-244X-9-26. 19473506 10.1186/1471-244X-9-26 PMC2701427

[56] Holländare F, Andersson G, Engström I. A comparison of psychometric properties between internet and paper versions of two depression instruments (BDI-II and MADRS-S) administered to clinic patients. J Med Internet Res. 2010, Dec, 19;12(5):e49. 10.2196/jmir.1392. 10.2196/jmir.1392 PMC3057311 21169165

[57] Berman AH, Bergman H, Palmstierna T, Schlyter F. Drug use disorders Identification Test [Internet]. 2016. Available from: https://doi.apa.org/doi/10.1037/t02890-000. cited 2024 Apr 12. .

[58] Hildebrand M. The psychometric properties of the drug use disorders Identification Test (DUDIT): a review of recent research. J Subst Abuse Treat. 2015, Jun;53:52–59. 10.1016/j.jsat.2015.01.008. 25682718 10.1016/j.jsat.2015.01.008

[59] Källmén H, Elgán TH, Wennberg P, Berman AH. Concurrent validity of the alcohol use disorders Identification Test (AUDIT) in relation to alcohol use disorder (AUD) severity levels according to the brief DSM-5 AUD diagnostic assessment screener. Nord J Psychiatry. 2019, Oct, 3;73(7):397–400. 10.1080/08039488.2019.1642382. 31347426 10.1080/08039488.2019.1642382

[60] Hagman BT. Development and psychometric analysis of the brief DSM-5 alcohol use disorder diagnostic assessment: towards effective diagnosis in college students. Phychol Addictive Behaviors. 2017, Nov;31(7):797–806. 10.1037/adb0000320. 10.1037/adb0000320 29144150

[61] Flannery BA, Volpicelli JR, Pettinati HM. Psychometric properties of the Penn alcohol craving scale. Alcohol Clin Exp Res. 1999, Aug;23(8):1289–95. 10.1111/j.1530-0277.1999.tb04349.x. 10470970

[62] American Psychiatric Association. Diagnostic and statistical manual of mental disorders: dSM-IV. 4. Washington, DC; 1998. p. 886 p, editor.

[63] Spitzer RL, Kroenke K, Williams JBW, Löwe B. A brief measure for assessing Generalized anxiety disorder: the GAD-7. Arch Intern Med. 2006, May, 22;166(10):1092. 10.1001/archinte.166.10.1092. 16717171 10.1001/archinte.166.10.1092

[64] Plummer F, Manea L, Trepel D, McMillan D. Screening for anxiety disorders with the GAD-7 and GAD-2: a systematic review and diagnostic metaanalysis. Gener Hosp Psychiatry. 2016, Mar;39:24–31. 10.1016/j.genhosppsych.2015.11.005. 10.1016/j.genhosppsych.2015.11.005 26719105

[65] DiClemente CC, Carbonari JP, Montgomery RP, Hughes SO. The alcohol abstinence self-efficacy scale. J Stud Alcohol. 1994, Mar;55(2):141–48. 10.15288/jsa.1994.55.141. 8189734 10.15288/jsa.1994.55.141

[66] Marlatt GA, George WH. Relapse prevention: introduction and overview of the model. Br J Addict. 1984, Sep;79(3):261–73. 10.1111/j.1360-0443.1984.tb00274.x. 6595020 10.1111/j.1360-0443.1984.tb00274.x

[67] Blomstrand A, Ariai N, Baar AC, Finbom-Forsgren BM, Thorn J, Björkelund C. Implementation of a low-budget, lifestyle-improvement method in an ordinary primary healthcare setting: a stepwise intervention study. BMJ Open. 2012;2(4):e001154. 10.1136/bmjopen-2012-001154. 10.1136/bmjopen-2012-001154 PMC3425908 22874629

[68] R Core Team. R: a language and environment for statistical Computing [internet]. 2023. Available from: https://www.R-project.org/. Vienna, Austria: R Foundation for Statistical Computing.

[69] Atkins DC, Baldwin SA, Zheng C, Gallop RJ, Neighbors C. A tutorial on count regression and zero-altered count models for longitudinal substance use data. Phychol Addictive Behaviors. 2013;27(1):166–77. 10.1037/a0029508. 10.1037/a0029508 PMC3513584 22905895

[70] Koller M. Robustlmm: an R package for robust estimation of linear mixed-effects models. J Stat Soft. 2016;75(6). 10.18637/jss.v075.i06.

[71] Brooks ME, Kristensen K, van Benthem KJ, Magnusson A, Berg CW, Nielsen A, et al. glmmTMB balances speed and flexibility among packages for zero-inflated generalized linear mixed modeling. R J. 2017;9(2):378–400. 10.32614/RJ-2017-066.

[72] Mallinckrodt CH, Clark WS, David SR. Accounting for dropout bias using mixed-effects models. J Biopharm Stat. 2001, Jan 1;11((1–2)):9–21. 10.1081/BIP-100104194. 11459446 10.1081/BIP-100104194

[73] Hallgren KA, Witkiewitz K. Missing data in alcohol clinical trials: a comparison of methods. Alcohol Clin Exp Res. 2013, Dec;37(12):2152–60. 10.1111/acer.12205. 23889334 10.1111/acer.12205 PMC4113114

[74] Mallinckrodt CH, Kaiser CJ, Watkin JG, Detke MJ, Molenberghs G, Carroll RJ. Type I error rates from likelihood-based repeated measures analyses of incomplete longitudinal data. Pharm Stat. 2004, Jul;3(3):171–86. 10.1002/pst.131.

[75] Chakraborty H. A mixed model approach for intent-to-treat analysis in longitudinal clinical trials with missing values [internet]. Research Triangle Park, NC: RTI Press; 2009 Apr. cited 2024 May 31: http://www.rti.org/publication/mixed-model-approach-intent-treat-analysis-longitudinal-clinical-trials-missing-values10.3768/rtipress.2009.mr.0009.0903. Report No. Available from. 30896910

[76] Peters SAE, Bots ML, Den Ruijter HM, Palmer MK, Grobbee DE, Crouse JR, et al. Multiple imputation of missing repeated outcome measurements did not add to linear mixed-effects models. J Retailing Clin Epidemiol. 2012, Jun;65(6):686–95. 10.1016/j.jclinepi.2011.11.012. 10.1016/j.jclinepi.2011.11.012 22459429

[77] Visser I, Speekenbrink M. depmixS4: an R package for hidden Markov models. J Stat Softw. 2010;36(7):1–21.

[78] Schulz KF, Altman DG, Consort MD. Statement: updated guidelines for reporting parallel group randomised trials. Ann Intern Med. 2010;152(11):726. 10.7326/0003-4819-152-11-201006010-00232. 2010 Jun 1. 20335313 10.7326/0003-4819-152-11-201006010-00232

[79] Sundström C, Eék N, Kraepelien M, Fahlke C, Gajecki M, Jakobson M, et al. High- versus low-intensity internet interventions for alcohol use disorders: results of a three-armed randomized controlled superiority trial. Addiction. 2020, May;115(5):863–74. 10.1111/add.14871. 31691413 10.1111/add.14871 PMC7187301

[80] Kypri K. Randomized controlled trial of web-based alcohol screening and brief intervention in primary care. Arch Intern Med. 2008, Mar, 10;168(5):530. 10.1001/archinternmed.2007.109. 18332300 10.1001/archinternmed.2007.109

[81] Riper H, Van Straten A, Keuken M, Smit F, Schippers G, Cuijpers P. Curbing problem drinking with personalized-feedback interventions. Am J Prev Med. 2009, Mar;36(3):247–55. 10.1016/j.amepre.2008.10.016. 19215850 10.1016/j.amepre.2008.10.016

[82] Walters GD. Spontaneous remission from alcohol, tobacco, and other drug abuse: seeking quantitative answers to qualitative questions. Am J Drug Alcohol Abuse. 2000, Jan;26(3):443–60. 10.1081/ADA-100100255. 10976668 10.1081/ada-100100255

[83] Kaner EF, Beyer FR, Garnett C, Crane D, Brown J, Muirhead C, et al. Cochrane Drugs and Alcohol Group. Personalised digital interventions for reducing hazardous and harmful alcohol consumption in community-dwelling populations. Cochrane Database Systematic Rev. 2017, Sep, 25;2017(9). 10.1002/14651858.CD011479.pub2 editor. 10.1002/14651858.CD011479.pub2 PMC6483779 28944453

[84] Gram IT, Larbi D, Wangberg SC. Comparing the efficacy of an identical, tailored smoking cessation intervention delivered by mobile text messaging versus email: randomized controlled trial. JMIR Mhealth Uhealth. 2019, Sep, 27;7(9):e12137. 10.2196/12137. 10.2196/12137 PMC6789425 31573935

[85] Keding A, Brabyn S, MacPherson H, Richmond SJ, Torgerson DJ. Text message reminders to improve questionnaire response rates. J Retailing Clin Epidemiol. 2016, Nov;79:90–95. 10.1016/j.jclinepi.2016.05.011. 10.1016/j.jclinepi.2016.05.011 27321820

[86] Severi E, Free C, Knight R, Robertson S, Edwards P, Hoile E. Two controlled trials to increase participant retention in a randomized controlled trial of mobile phone-based smoking cessation support in the United Kingdom. Clin Trials. 2011, Oct;8(5):654–60. 10.1177/1740774511416524. 21933834 10.1177/1740774511416524 PMC3573670

