# Supplementary material for: App-based skills training to reduce problem drinking among adult internet help-seekers: a double-blinded randomized controlled trial
Source: Addict Sci Clin Pract. 2026 Mar 31;21:33. doi: 10.1186/s13722-026-00663-5 (PMC13041508; doi:10.1186/s13722-026-00663-5)

**Supplemental Table 1**

Results for Secondary Hypothesis 4

Summary of chi-squared tests for excessive drinking by timepoint and group (DDQ-old)

| Timepoint | Chi-squared statistic | *p* |
| --- | --- | --- |
| Baseline | 1.00 | 0.32 |
| 6 weeks | 0.24 | 0.63 |
| 12 weeks | 0.24 | 0.63 |
| 26 weeks | 0.95 | 0.33 |

Summary of chi-squared tests for excessive drinking by timepoint and group (DDQ-new)

| Timepoint | Chi-squared statistic | *p* |
| --- | --- | --- |
| Baseline | 0.01 | 0.91 |
| 6 weeks | 0.60 | 0.44 |
| 12 weeks | 0.28 | 0.60 |
| 26 weeks | 0.74 | 0.39 |

Summary of chi-squared tests for excessive drinking by timepoint and group (TLFB-old)

| Timepoint | Chi-squared statistic | *p* |
| --- | --- | --- |
| Baseline | 0.00 | 1.00 |
| 6 weeks | 1.17 | 0.28 |
| 12 weeks | 0.89 | 0.35 |
| 26 weeks | 1.06 | 0.30 |

Summary of chi-squared tests for excessive drinking by timepoint and group (TLFB-new)

| Timepoint | Chi-squared statistic | *p* |
| --- | --- | --- |
| Baseline | 0.00 | 1.00 |
| 6 weeks | 0.79 | 0.37 |
| 12 weeks | 0.26 | 0.61 |
| 26 weeks | 1.04 | 0.30 |

**Supplemental Table 2**Valid Follow-up Completion

| Time | TeleCoach | | |  | Control | | |  |  | Between-group analysis | | |
| --- | --- | --- | --- | --- | --- | --- | --- | --- | --- | --- | --- | --- |
|  | *N* | % |  |  | *N* | % |  |  |  | χ² | *df* | *p* |
| 6-week follow-up | 187 | 64% |  |  | 190 | 67% |  |  |  | 0.56 | 1 | .45 |
| 12-week follow-up | 159 | 54% |  |  | 172 | 61% |  |  |  | 2.24 | 1 | .14 |
| 36-week follow-up | 138 | 47% |  |  | 151 | 53% |  |  |  | 2.01 | 1 | .16 |

**Supplemental Table 3**Results from Zero-Inflated Negative Binomial Regression (ZINB)

ZINB Estimates for the Primary Hypothesis. Significant estimates marked in bold type.

| Variable | Incidence Rate Ratio | *CI* | *p* |  |
| --- | --- | --- | --- | --- |
| **Count Model** | | | | |
| (Intercept) | 14.39 | 11.48 – 18.03 | **<0.001** |  |
| TeleCoach | 1.04 | 0.94 – 1.15 | 0.466 |  |
| 6-week follow-up | 0.61 | 0.55 – 0.67 | **<0.001** |  |
| 12-week follow-up | 0.57 | 0.52 – 0.63 | **<0.001** |  |
| 26-week follow-up | 0.58 | 0.52 – 0.64 | **<0.001** |  |
| Age | 1.01 | 1.00 – 1.01 | **0.003** |  |
| Gender – Male | 1.35 | 1.23 – 1.48 | **<0.001** |  |
| Sought help before | 1.14 | 1.03 – 1.26 | **0.010** |  |
| GAD 7 | 1.00 | 0.99 – 1.01 | 0.888 |  |
| MADRS | 1.01 | 1.00 – 1.02 | **0.004** |  |
| 6-week * TeleCoach | 1.02 | 0.89 – 1.17 | 0.751 |  |
| 12-week * TeleCoach | 1.06 | 0.92 – 1.22 | 0.456 |  |
| 26-week * TeleCoach | 0.92 | 0.79 – 1.07 | 0.265 |  |
| **Zero-Inflated Model** | | | |  |
| (Intercept) | 0.00 | 0.00 – 0.00 | **<0.001** |  |
| TeleCoach | 1.14 | 0.13 – 10.28 | 0.905 |  |
| 6-week follow-up | 35.49 | 6.25 – 201.63 | **<0.001** |  |
| 12-week follow-up | 88.10 | 14.55 – 533.58 | **<0.001** |  |
| 26-week follow-up | 45.34 | 7.86 – 261.40 | **<0.001** |  |
| Age | 1.00 | 0.95 – 1.05 | 1.000 |  |
| Gender – Male | 1.67 | 0.56 – 4.95 | 0.358 |  |
| Sought help before | 4.25 | 1.33 – 13.56 | **0.015** |  |
| GAD 7 | 1.04 | 0.92 – 1.16 | 0.553 |  |
| MADRS | 0.99 | 0.92 – 1.08 | 0.880 |  |
| 6-week * TeleCoach | 2.76 | 0.31 – 24.81 | 0.364 |  |
| 12-week * TeleCoach | 0.64 | 0.07 – 5.85 | 0.696 |  |
| 26-week * TeleCoach | 1.13 | 0.12 – 10.65 | 0.916 |  |
| **Random Effects** | | | |  |
| σ^2^ | 0.29 | | |  |
| τ_00_ _Participant_ | 0.17 | | |  |
| ICC | 0.37 | | |  |
| N _Participant_ | 573 | | |  |
| Observations | 1513 | | |  |
| Marginal R^2^ / Conditional R^2^ | 0.188 / 0.490 | | |  |

**Note.** Estimates were reported as contrasts; the intercept indicates the estimate for the control group, when the influence of time, covariates, and interactions were set to zero.

ZINB Estimates for Secondary hypothesis 1. Significant estimates marked in bold type.

| Variable | Incidence Rate Ratio | *CI* | *p* |  |
| --- | --- | --- | --- | --- |
| **Count Model** | | | | |
| (Intercept) | 13.73 | 10.05 – 18.75 | **<0.001** |  |
| 6-week follow-up | 0.80 | 0.56 – 1.14 | 0.214 |  |
| 12-week follow-up | 1.15 | 0.80 – 1.64 | 0.459 |  |
| 26-week follow-up | 0.87 | 0.61 – 1.24 | 0.440 |  |
| Motivation to reduce | 1.01 | 0.98 – 1.03 | 0.677 |  |
| Age | 1.01 | 1.00 – 1.01 | **0.002** |  |
| Gender – Male | 1.35 | 1.23 – 1.48 | **<0.001** |  |
| Sought help before | 1.13 | 1.02 – 1.25 | **0.015** |  |
| GAD 7 | 1.00 | 0.99 – 1.01 | 0.904 |  |
| MADRS | 1.01 | 1.00 – 1.02 | **0.003** |  |
| 6-week * Motivation | 0.97 | 0.93 – 1.01 | 0.128 |  |
| 12-week * Motivation | 0.92 | 0.89 – 0.96 | **<0.001** |  |
| 26-week * Motivation | 0.95 | 0.91 – 0.99 | **0.012** |  |
| **Zero-Inflated Model** | | | |  |
| (Intercept) | 0.00 | 0.00 – 0.00 | **<0.001** |  |
| 6-week follow-up | 64.70 | 0.12 – 34782.27 | 0.194 |  |
| 12-week follow-up | 884.66 | 1.57 – 497679.24 | **0.036** |  |
| 26-week follow-up | 877.24 | 1.53 – 504501.96 | **0.037** |  |
| Motivation to reduce | 1.54 | 0.79 – 3.00 | 0.200 |  |
| Age | 1.00 | 0.95 – 1.04 | 0.857 |  |
| Gender – Male | 1.80 | 0.64 – 5.11 | 0.268 |  |
| Sought help before | 4.43 | 1.44 – 13.63 | **0.010** |  |
| GAD 7 | 1.03 | 0.92 – 1.15 | 0.586 |  |
| MADRS | 1.00 | 0.92 – 1.08 | 0.943 |  |
| 6-week * Motivation | 0.98 | 0.50 – 1.90 | 0.948 |  |
| 12-week * Motivation | 0.74 | 0.38 – 1.44 | 0.381 |  |
| 26-week * Motivation | 0.71 | 0.37 – 1.39 | 0.323 |  |
| **Random Effects** | | | |  |
| σ^2^ | 0.28 | | |  |
| τ_00_ _Participant_ | 0.17 | | |  |
| ICC | 0.37 | | |  |
| N _Participant_ | 573 | | |  |
| Observations | 1513 | | |  |
| Marginal R^2^ / Conditional R^2^ | 0.196 / 0.497 | | |  |

**Note.** Estimates were reported as contrasts; the intercept indicates the estimate for the control group, when the influence of time, covariates, and interactions were set to zero.

ZINB Estimates for the Secondary Hypothesis 2. Significant estimates marked in bold type.

| Variable | Incidence Rate Ratio | *CI* | *p* |  |
| --- | --- | --- | --- | --- |
| **Count Model** | | | | |
| (Intercept) | 11.70 | 7.91 – 17.32 | **<0.001** |  |
| 6-week follow-up | 0.84 | 0.51 – 1.39 | 0.507 |  |
| 12-week follow-up | 0.93 | 0.57 – 1.52 | 0.780 |  |
| 26-week follow-up | 0.89 | 0.54 – 1.49 | 0.668 |  |
| TeleCoach | 1.39 | 0.85 – 2.28 | 0.185 |  |
| Motivation to reduce | 1.02 | 0.98 – 1.06 | 0.250 |  |
| Age | 1.01 | 1.00 – 1.01 | **0.002** |  |
| Gender – Male | 1.35 | 1.23 – 1.48 | **<0.001** |  |
| Sought help before | 1.13 | 1.02 – 1.25 | **0.015** |  |
| GAD 7 | 1.00 | 0.99 – 1.01 | 0.909 |  |
| MADRS | 1.01 | 1.00 – 1.02 | **0.003** |  |
| 6-week * TeleCoach | 0.90 | 0.45 – 1.81 | 0.770 |  |
| 12-week * TeleCoach | 1.57 | 0.76 – 3.24 | 0.224 |  |
| 26-week * TeleCoach | 0.98 | 0.48 – 2.00 | 0.951 |  |
| 6-week * Motivation | 0.96 | 0.91 – 1.02 | 0.187 |  |
| 12-week * Motivation | 0.94 | 0.89 – 1.00 | **0.044** |  |
| 26-week * Motivation | 0.95 | 0.90 – 1.01 | 0.086 |  |
| TeleCoach * Motivation | 0.97 | 0.91 – 1.02 | 0.231 |  |
| 6-week * TeleCoach * Motivation | 1.01 | 0.94 – 1.10 | 0.731 |  |
| 12-week * TeleCoach * Motivation | 0.95 | 0.88 – 1.04 | 0.250 |  |
| 26-week * TeleCoach * Motivation | 0.99 | 0.91 – 1.08 | 0.816 |  |
| **Zero-Inflated Model** | | | |  |
| (Intercept) | 0.00 | 0.00 – 0.01 | **0.003** |  |
| 6-week follow-up | 0.62 | 0.00 – 12213.54 | 0.926 |  |
| 12-week follow-up | 25.40 | 0.02 – 30522.92 | 0.371 |  |
| 26-week follow-up | 281.25 | 0.13 – 610227.80 | 0.150 |  |
| TeleCoach | 0.01 | 0.00 – 171661.27 | 0.606 |  |
| Motivation to reduce | 1.37 | 0.61 – 3.09 | 0.451 |  |
| Age | 0.99 | 0.95 – 1.04 | 0.783 |  |
| Gender – Male | 1.79 | 0.60 – 5.33 | 0.299 |  |
| Sought help before | 4.70 | 1.47 – 15.05 | **0.009** |  |
| GAD 7 | 1.03 | 0.92 – 1.16 | 0.602 |  |
| MADRS | 0.99 | 0.92 – 1.07 | 0.866 |  |
| 6-week * TeleCoach | 6968.25 | 0.00 – 274018929815.81 | 0.321 |  |
| 12-week * TeleCoach | 8242.42 | 0.00 – 86244975712.13 | 0.274 |  |
| 26-week * TeleCoach | 120.34 | 0.00 – 1439736444.13 | 0.565 |  |
| 6-week * Motivation | 1.52 | 0.54 – 4.31 | 0.430 |  |
| 12-week * Motivation | 1.13 | 0.53 – 2.42 | 0.744 |  |
| 26-week * Motivation | 0.81 | 0.36 – 1.83 | 0.610 |  |
| TeleCoach * Motivation | 1.61 | 0.28 – 9.07 | 0.591 |  |
| 6-week * TeleCoach * Motivation | 0.44 | 0.07 – 2.76 | 0.378 |  |
| 12-week * TeleCoach * Motivation | 0.35 | 0.06 – 1.94 | 0.230 |  |
| 26-week * TeleCoach * Motivation | 0.60 | 0.11 – 3.39 | 0.567 |  |
| **Random Effects** | | | |  |
| σ^2^ | 0.28 | | |  |
| τ_00_ _Participant_ | 0.17 | | |  |
| ICC | 0.37 | | |  |
| N _Participant_ | 573 | | |  |
| Observations | 1513 | | |  |
| Marginal R^2^ / Conditional R^2^ | 0.199 / 0.496 | | |  |

**Note.** Estimates were reported as contrasts; the intercept indicates the estimate for the control group, when the influence of time, covariates, and interactions were set to zero.

**Supplemental Figure 1**Histograms of participant clinical characteristics by group


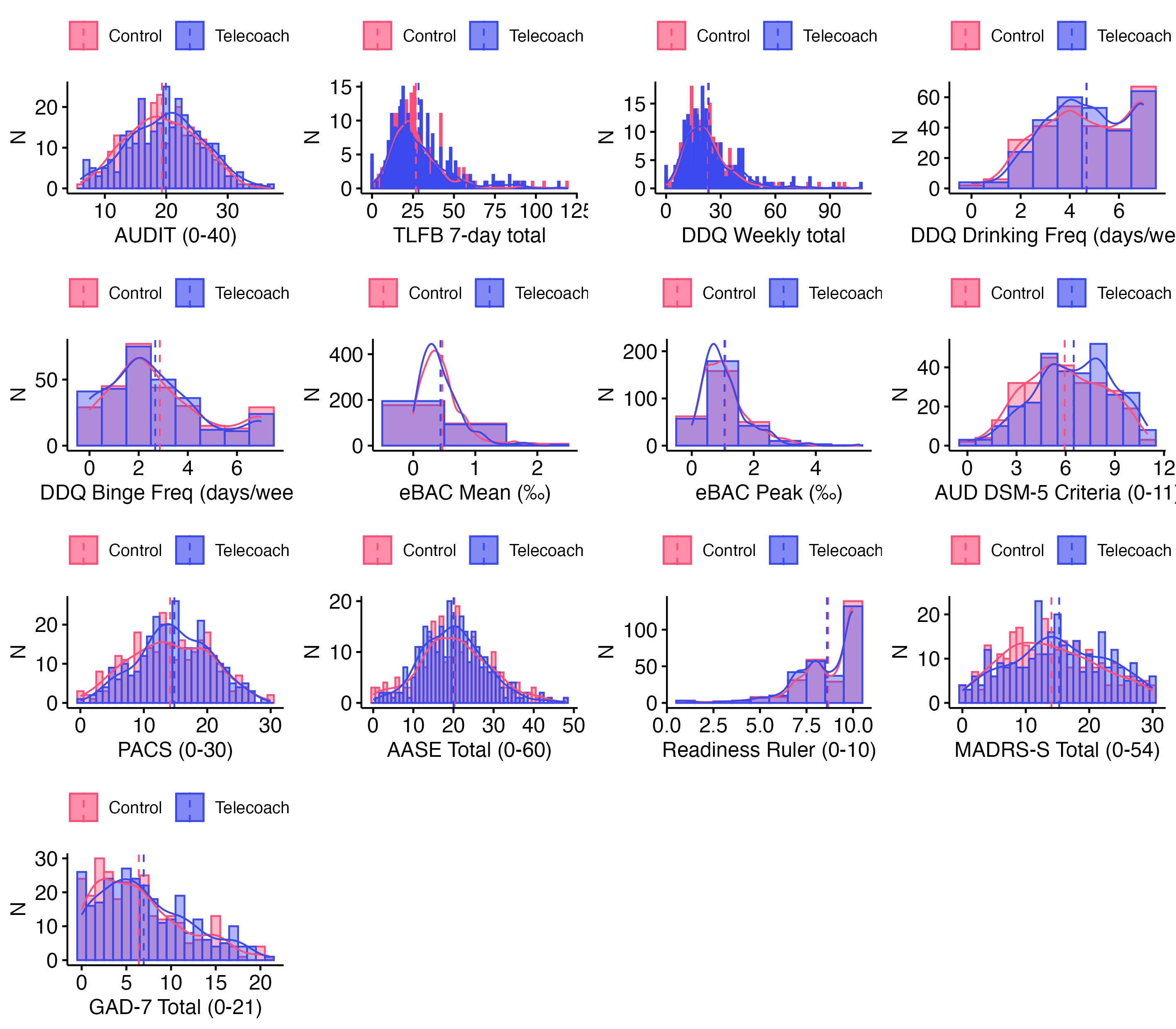


**Supplemental Figure 2**

Additional visualizations for HMM results

Illustration of participant flow based on state transition probabilities.


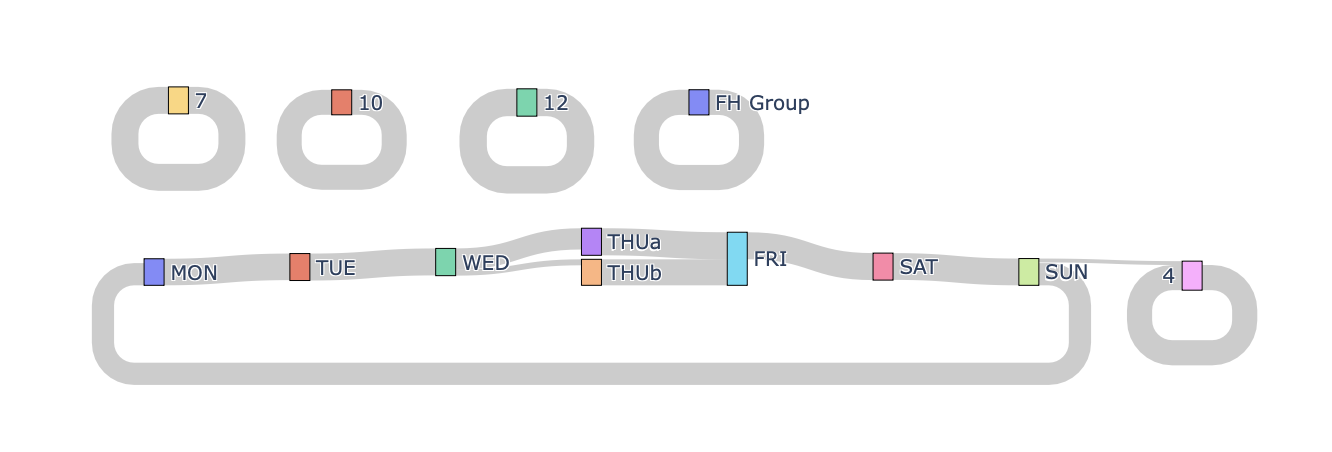
For visual clarity, only transition probability >0.05 is shown here.

Transition matrix for the 13-state Hidden Markov Model.

**
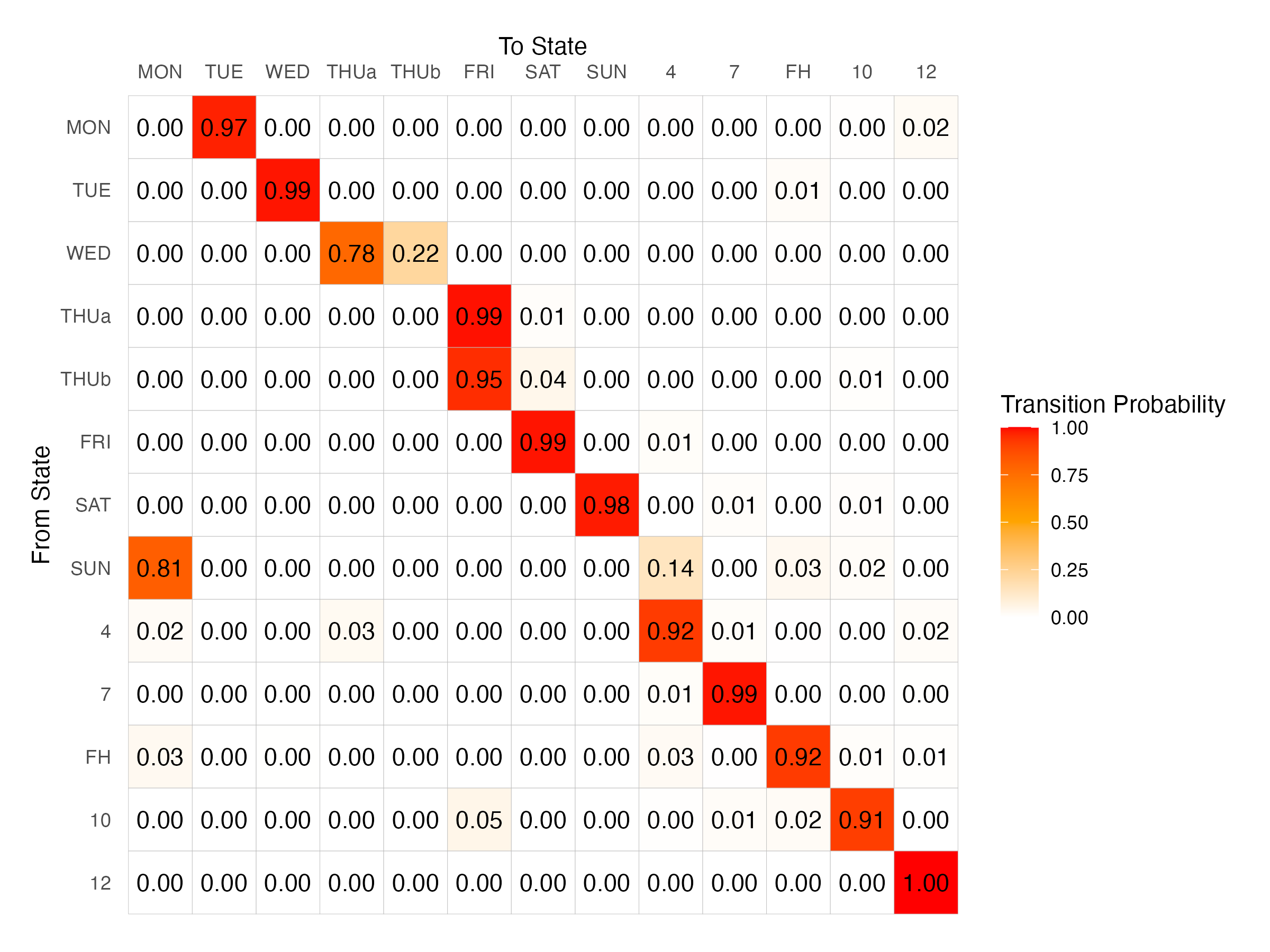
**

**Supplemental Figure 3**
Mean visits to each content unit per-user in the intervention app.


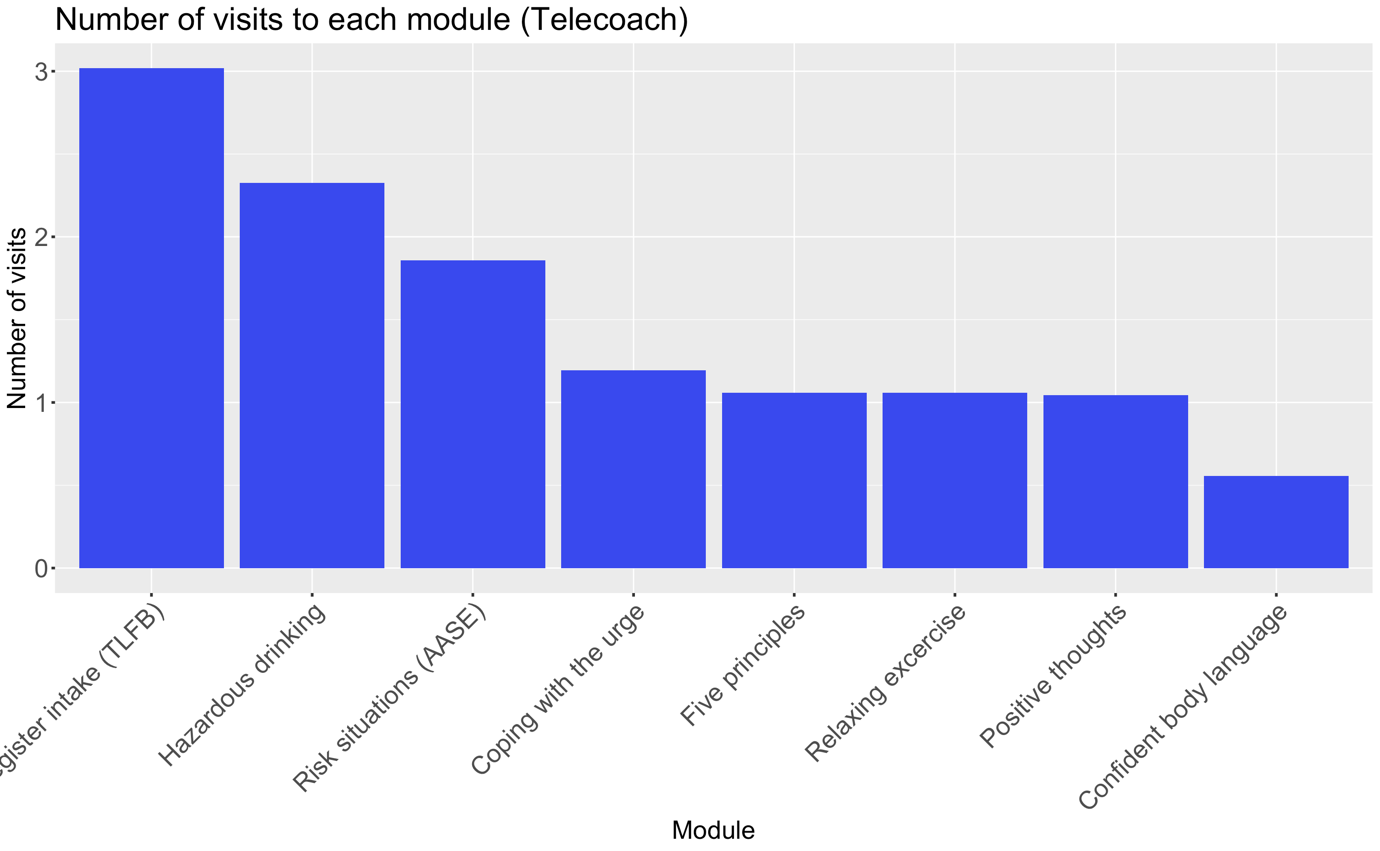


**Supplemental Figure 4**
Mean visits to each content unit per user in the control app.


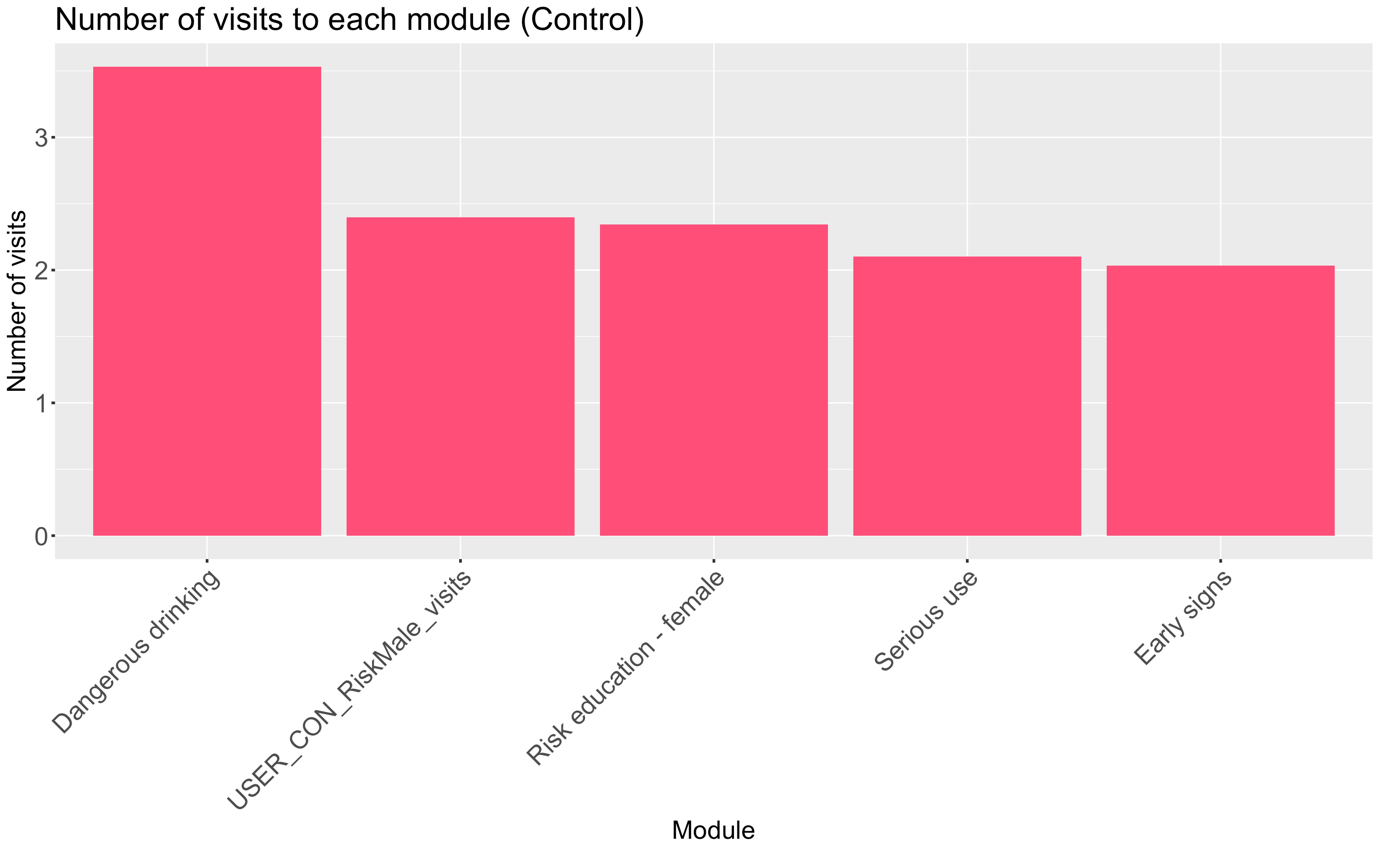

Supplement: Supplementary file 1 — Supplementary material 1 [file 13722_2026_663_MOESM1_ESM.docx]
